# Molecular determinants for differential activation of the bile acid receptor from the pathogen *Vibrio parahaemolyticus*

**DOI:** 10.1016/j.jbc.2023.104591

**Published:** 2023-03-07

**Authors:** Angela J. Zou, Lisa Kinch, Suneeta Chimalapati, Nalleli Garcia, Diana R. Tomchick, Kim Orth

**Affiliations:** 1 Department of Molecular Biology, University of Texas Southwestern Medical Center, Dallas, Texas, USA; 2 Department of Biochemistry, University of Texas Southwestern Medical Center, Dallas, Texas, USA; 3 Howard Hughes Medical Institute, University of Texas Southwestern Medical Center, Dallas, Texas, USA; 4 Department of Microbiology and Cell Science, University of Florida Institute of Food and Agricultural Sciences, Gainesville, Florida, USA; 5 Department of Biophysics, University of Texas Southwestern Medical Center, Dallas, Texas, USA

**Keywords:** bile acid, receptor structure–function, type III secretion system, bacterial pathogenesis, host–pathogen interaction

## Abstract

Bile acids are important for digestion of food and antimicrobial activity. Pathogenic *Vibrio parahaemolyticus* senses bile acids and induce pathogenesis. The bile acid taurodeoxycholate (TDC) was shown to activate the master regulator, *VtrB*, of this system, whereas other bile acids such as chenodeoxycholate (CDC) do not. Previously, VtrA–VtrC was discovered to be the co-component signal transduction system that binds bile acids and induces pathogenesis. TDC binds to the periplasmic domain of the VtrA–VtrC complex, activating a DNA-binding domain in VtrA that then activates *VtrB.* Here, we find that CDC and TDC compete for binding to the VtrA–VtrC periplasmic heterodimer. Our crystal structure of the VtrA–VtrC heterodimer bound to CDC revealed CDC binds in the same hydrophobic pocket as TDC but differently. Using isothermal titration calorimetry, we observed that most mutants in the binding pocket of VtrA–VtrC caused a decrease in bile acid binding affinity. Notably, two mutants in VtrC bound bile acids with a similar affinity as the WT protein but were attenuated for TDC-induced type III secretion system 2 activation. Collectively, these studies provide a molecular explanation for the selective pathogenic signaling by *V. parahaemolyticus* and reveal insight into a host’s susceptibility to disease.

*Vibrio parahaemolyticus* is a gram-negative pathogen that is a leading cause of gastroenteritis from the consumption of contaminated raw seafood around the world (1). Many cellular factors contribute to the virulence of *V. parahaemolyticus*, including two thermostable hemolysins and two type III secretion systems (T3SSs), known as T3SS1 and T3SS2 (1). The T3SSs are large needle-like protein complexes that transport bacterial effector proteins, known as Vops, from the bacterial cytoplasm into the host cell cytoplasm to promote infection (1). The T3SS2 is found only in clinical isolates, and its presence coincides with gastroenteritis as well as with bacterial invasion and intracellular replication in the host gut epithelium (1, 2, 3). T3SS2 expression is regulated by VtrA–VtrC, a member of a newly described superfamily of bacterial “co-component” signaling systems (4). VtrA–VtrC respond to bile acids in the host intestinal tract and induce expression of VtrB, another inner membrane transcription factor. VtrB then induces expression of the T3SS2 (5, 6).

The newly described VtrA–VtrC-like superfamily of co-component signaling systems in enteric pathogens include members like ToxR/ToxS and TcpP/TcpH in *Vibrio cholerae* and PsaE/PsaF in *Yersinia pestis* (4). Members of this family share several characteristics where the components of these signaling systems adopt similar domain organizations. The transcription factor component (VtrA) includes an N-terminal helix–turn–helix DNA-binding domain (DBD), a single transmembrane helix, and a C-terminal periplasmic domain, whereas the sensor component (VtrC) includes a transmembrane helix followed by a lipocalin-like periplasmic domain. The co-component-encoding genes share a similar operon arrangement, and their gene products form, or are predicted to form, an obligate heterodimer *via* interaction of the periplasmic domains (4, 7). In the case of VtrA–VtrC, VtrA functions as a membrane-bound transcription factor, whereas VtrC in complex with VtrA senses bile. When VtrA–VtrC bind bile acids in the periplasm, the VtrA DBD is activated in the cytoplasm (7, 8). These characteristics distinguish co-component membrane signaling systems from a subset of membrane-tethered one-component systems exemplified by the *Escherichia coli* pH sensor CadC (4, 9) as well as from two-component systems requiring an intracellular histidine kinase for signaling (10).

Previously, *V. parahaemolyticus* VtrA–VtrC was shown to turn on T3SS2-mediated virulence by sensing the presence of host intestinal bile acids (6, 7). However, certain types of bile acids such as taurodeoxycholate (TDC) induce *VtrB* expression and T3SS2 activation, but others such as chenodeoxycholate (CDC) do not (6, 7). The molecular mechanism behind the variable activation of *VtrB* expression by bile acids is unknown. Here, we use isothermal titration calorimetry (ITC) to show that the bile acid CDC binds to the VtrA–VtrC periplasmic complex with a similar affinity as with TDC. We then solved the structure of the VtrA–VtrC complex bound with CDC and observed that CDC binds in the same hydrophobic pocket of the VtrA–VtrC complex as TDC. Interestingly, the two different bile acids compete to induce or suppress *VtrB* expression. Further analysis revealed that TDC and CDC make different sets of interactions with the VtrA–VtrC barrel. Many mutations of residues within the bile acid–binding pocket disrupt binding of TDC and CDC to VtrA–VtrC as well as *VtrB* expression activated by TDC. However, one mutant in VtrC, S123A, did not disrupt binding but altered signaling, and the H50A mutant had marginal effects on binding, yet muted signaling. These results provide a molecular explanation for the opposing outcomes TDC and CDC have on inducing *VtrB* expression and the pathogenic T3SS2.

## Results and discussion

### CDC binds to VtrA–VtrC and competes with TDC

**Figure 1:**
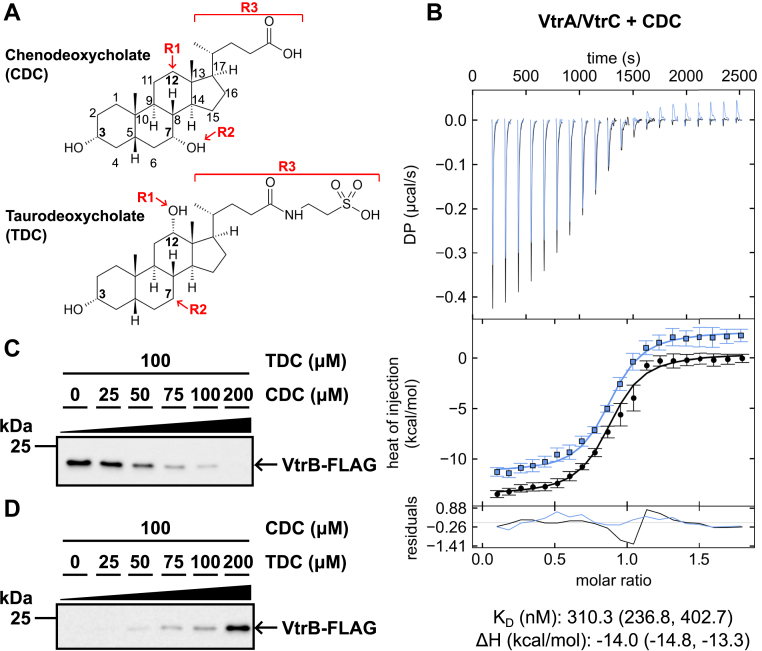
**CDC binds to VtrA–VtrC and competes with TDC.***A*, chemical structures of CDC (*top*) and TDC (*bottom*). *Numbers* denote positions of carbon atoms in the steroid nucleus. *Bold numbers* indicate carbon atoms that have an attached hydroxyl group in TDC and/or CDC. *Red text* and *arrows* indicate differences between CDC and TDC at positions R1 and R2 as well as the length of the side chain (R3). Images were created in ChemSketch. *B*, ITC thermograms for CDC binding to the VtrA–VtrC periplasmic domain complex. Thermodynamic parameters were determined by global fitting of duplicate isotherms (presented in *black* and *blue*). The dissociation constant (*K*_*D*_) and enthalpy (ΔH) values are reported with 1σ error intervals in parenthesis. *C*, Western blot analysis of CDC/TDC competition assay. *Vibrio parahaemolyticus* was grown in media supplemented with 100 μM TDC and varying concentrations of CDC from 0 to 200 μM. Anti-FLAG antibody (Sigma) was used to detect FLAG-VtrB. *D*, Western blot analysis of TDC–CDC competition assay. Experiment was performed as in (*C*), except *V. parahaemolyticus* was grown in media supplemented with 100 μM CDC and varying concentrations of TDC from 0 to 200 μM. Results in (*C* and *D*) are representative of three independent experiments. CDC, chenodeoxycholate; ITC, isothermal titration calorimetry; TDC, taurodeoxycholate.

The chemical structures of the bile acids TDC and CDC share a common steroid nucleus containing three six-membered rings fused to a fourth five-membered ring (Fig. 1*A*). Attached to the steroid nucleus are a hydroxyl group at position 3α (3α-OH) and a five carbon side chain attached to the five-membered ring. The structures of TDC and CDC differ in the position of an additional hydroxyl group attached to the steroid nucleus. TDC has a second hydroxyl group at position 12α (R1, Fig. 1*A*), whereas CDC has a second hydroxyl group at position 7α (R2, Fig. 1*A*). The carbon side chain of CDC ends with a carboxylic acid and contains five carbon atoms (R3, Fig. 1*A*). Meanwhile, the carbon side chain of TDC is conjugated to a taurine residue *via* N-acyl amidation, resulting in a longer side chain with seven carbon atoms (R3, Fig. 1*A*) (11). To distinguish the different lengths of the side chains in CDC and TDC, the R3 group will be referred to as “5Tail” for CDC and “7Tail” for TDC. Because of the similarities in the structures of TDC and CDC, we reasoned that CDC can bind the same hydrophobic pocket in VtrA–VtrC as bound by TDC. However, differences in the position of the hydroxyl groups and the length of the side chains in TDC and CDC may lead to different interactions with VtrA–VtrC and thus opposing outcomes in *VtrB* transcription initiation.

We tested whether CDC binds to the VtrA–VtrC periplasmic domain complex using ITC. Like TDC (7), negative power deflections were seen throughout titration of CDC into the VtrA–VtrC solution, indicating CDC binds to the complex in an exothermic manner (Fig. 1*B*). The *K*_*D*_ of the CDC and VtrA–VtrC interaction was 310.3 nM with a molar ratio of approximately 1:1 (n = 0.85) (Fig. 1*B* and Table 1). The previously reported *K*_*D*_ of the TDC and VtrA–VtrC interaction was 315.4 nM (7). When we repeated the ITC experiment with TDC and VtrA–VtrC, we obtained a lower *K*_*D*_ of 129.4 nM (Table 1 and Fig. S7). In this study, CDC bound to VtrA–VtrC with a weaker affinity compared with TDC.

**Table 1:** Thermodynamic parameters of TDC and CDC binding with various VtrA–VtrC constructs determined by ITC

VtrC construct	Bile acid	ΔG (kcal/mol)	ΔH (kcal/mol)	ΔS (cal/mol∗K)	TΔS (kcal/mol)	*K*_*D*_ (nM)	Fold change in affinity compared with WT
WT	TDC	−9.2	−11.4	−7.4	−2.2	129.4	—
H50A	TDC	−8.7	−11.7	−10.2	−3.0	301.2	Twofold
Y81A	TDC	−7.9	−6.6	4.6	1.3	1189.1	Ninefold
S123A	TDC	−9.4	−9.1	1.0	0.3	91.2	No change
Y151A	TDC	−7.5	−11.6	−13.9	−4.1	2499.7	19-fold
Y151F	TDC	−8.1	−10.2	−7.2	−2.1	873.2	Sevenfold
							
WT	CDC	−8.7	−14.0	−17.8	−5.2	310.3	—
H50A	CDC	−8.1	−9.2	−3.9	−1.1	904.5	Threefold
Y81A	CDC	−8.2	−7.8	1.5	0.4	730.7	Twofold
S123A	CDC	−9.0	−13.9	−16.7	−4.9	202.1	No change
Y151A	CDC	−7.6	−13.9	−21.7	−6.4	2282.9	Sevenfold
Y151F	CDC	−8.0	−9.6	−5.6	−1.6	1166.3	Fourfold

Gotoh *et al.* (6) previously demonstrated that TDC and CDC have disparate effects on the activation of *VtrB*. Since we observed that both CDC and TDC bind to VtrA–VtrC, we tested whether the two bile acids can compete to activate *VtrB* expression. We used an attenuated *V. parahaemolyticus* clinical RimD2210633 derivative strain POR1 that is deleted for hemolysins (*ΔtdhAS*) and contains a 3XFLAG tag inserted at the 3′ end of the native *VtrB* open reading frame. We grew this strain in media supplemented with 100 μM TDC and varying concentrations of CDC from 0 to 200 μM. Western blot analysis of *VtrB*-FLAG expression revealed *VtrB* is expressed when treated with 100 μM TDC alone. However, *VtrB* expression gradually decreased with the addition of increasing amounts of CDC (Fig. 1*C*). In the reverse experiment, *V. parahaemolyticus* was grown in media supplemented with 100 μM CDC and varying concentrations of TDC from 0 to 200 μM. *VtrB* was not expressed when treated with 100 μM CDC alone. *VtrB* expression gradually increased with the addition of increasing amounts of TDC (Fig. 1*D*). These results indicate that TDC and CDC compete to induce or suppress *VtrB* expression, respectively.

### CDC binds to VtrA–VtrC in the same hydrophobic pocket as TDC

**Figure 2:**
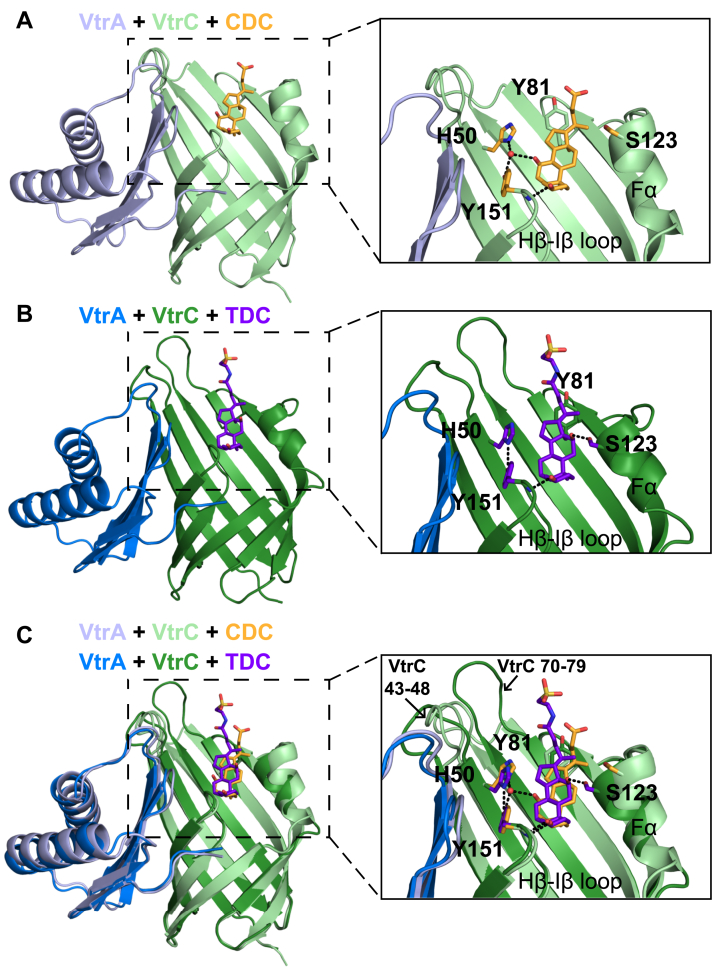
**The bile acids CDC and TDC make different sets of interactions with the VtrC periplasmic domain.***A*, structure of the periplasmic domain complex formed by VtrA (*light blue*) and VtrC (*light green*) bound to CDC (*orange*). View of the CDC binding site shown in the *box* to the *right* with interacting residues shown as *sticks* and a coordinated water molecule represented as a *red sphere*. Hydrogen bonds are represented as *dashed lines*. *B*, structure of the VtrA (*dark blue*) and VtrC (*dark green*) complex bound to TDC (*purple*). View of the TDC binding site shown in the *box* to the *right* with the same residues as in (*A*) shown as *sticks*. *C*, overlay of the images in (*A* and *B*) showing differences in the structures of VtrA–VtrC bound to CDC and TDC. Images in (*A*–*C*) were generated from the structures of the CDC- and TDC-bound heterodimers with the closest mean temperature factors to each other (Table S1). CDC, chenodeoxycholate; TDC, taurodeoxycholate.

The competitive effects of TDC and CDC on *VtrB* expression suggest these bile acids bind to the same region of VtrA–VtrC. We purified the VtrA–VtrC periplasmic domain complex in the presence of CDC, crystallized this complex in space group C2, and obtained the X-ray structure. The crystal structure was solved using molecular replacement with the TDC-bound structure (Protein Data Bank [PDB] ID: 5KEW, space group P2_1_2_1_2_1_) as a search model and refined to a resolution of 2.08 Å. The CDC-bound VtrA–VtrC asymmetric unit contained four VtrA–VtrC heterodimers, each bound with one CDC molecule inside the β-barrel (Figs. 2*A* and S1).

Despite their common steroid nucleus, CDC is modified with an R1-OH that is absent in TDC (Fig. 1*A*). This unique CDC hydroxyl group forms a hydrogen bond with a water molecule that is also coordinated by VtrC H50 ND1 and Y151-OH (Fig. 2*A*). The backbone amide of VtrC Y151 forms a hydrogen bond with the 3α-OH of CDC, and VtrC Y81 makes van der Waals contacts with CDC. Electron density for CDC and the surrounding binding pocket residues was more defined in two of the four VtrA–VtrC heterodimers (Fig. S1). The models for the heterodimers with lower atomic displacement parameters (ADPs) and more defined density included a water molecule in the binding pocket, whereas the other heterodimers did not. However, the distance between H50 ND1 and Y151-OH was very similar in all four heterodimers, ranging from 4.7 to 5.0 Å. The CDC binding site overlaps with the previously determined TDC site, with the R3-7Tail facing outside the barrel (Fig. 2*B*), The steroid backbone of each bile acid overlaps in a superposition of the two structures, with the R3-7Tail of TDC exhibiting the largest deviation (Fig. 2*C*). The relative position of VtrA is similar in both structures.

**Figure 3:**
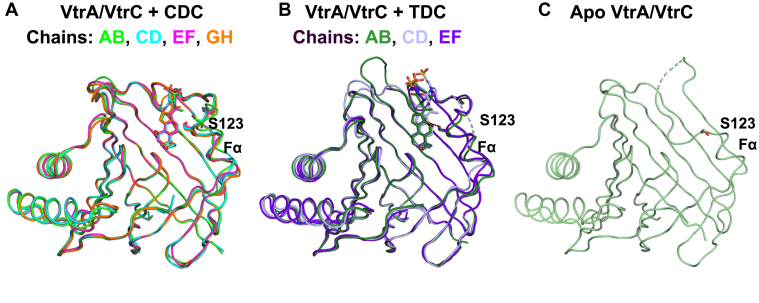
**Comparison of bile acid–bound and apo crystal structures**. *A*, superposition of all four heterodimers in the asymmetric unit of the CDC-bound crystal. *B*, all three heterodimers in the asymmetric unit of the TDC-bound crystal. *C*, single heterodimer in the asymmetric unit of the apo crystal. Structures of VtrA–VtrC are represented by *ribbons*. CDC, TDC, and S123 are shown as *sticks*. S123 and the Fα helix are labeled. Hydrogen bond between TDC and S123 in each heterodimer is shown as *dashed lines*. CDC, chenodeoxycholate; TDC, taurodeoxycholate.

Alignment of all four CDC-bound heterodimers in the asymmetric unit showed a difference in the position of the short VtrC helix Fα in one heterodimer compared with the other heterodimers (Figs. 3*A* and S2*A*). Fα was located further from the CDC binding site in one heterodimer (chains A/B). This helix forms direct contacts with a symmetry mate in the crystal lattice. In the other three heterodimers, Fα does not contact symmetry mates. Meanwhile, on the other side of the VtrA–VtrC β-barrel, the VtrC Hβ–Iβ loop forms part of the interface with VtrA (Fig. 2*A*). Within this loop is VtrC Y151, which forms a hydrogen bond with CDC 3α-OH. Residue Y151 is aligned closely near the VtrA interface in all four CDC-bound heterodimers (Fig. S2*A*). Also near the VtrA–VtrC interface is a flexible loop containing VtrC residues 43 to 48 (Fig. S2*A*). The backbone of this loop adopts similar positions in all four CDC-bound heterodimers, positioning Y48 toward the CDC binding site and D45 near the VtrA interface (Fig. S2*C*). The RMSD of VtrA among the four heterodimers ranged from 0.3 to 0.8 Å, whereas the RMSD of VtrC ranged from 0.5 to 0.8 Å (Fig. S2*A*, *right panel*). VtrA and VtrC in chains A and B had the largest RMSDs compared with the other three heterodimers, ranging from 0.7 to 0.8 Å for each.

### Binding pocket changes are observed with CDC or TDC and VtrA–VtrC

The previously determined TDC-bound structure revealed a key interaction with VtrC S123 that ordered part of the helix Fα in the presence of ligand (7). In the TDC-bound structure, the R1-OH of TDC makes a hydrogen bond with VtrC S123-OH (Figs. 1*A* and 2*B*). VtrC H50-ND1 and Y151-OH form a direct hydrogen bond, instead of coordinating a water molecule as is observed in the CDC-bound structure (Fig. 2). The backbone amide of Y151 forms a hydrogen bond with TDC 3α-OH, similar to that observed in CDC. Y81 forms van der Waals contacts with the carbon side chain from TDC.

A superposition of the TDC- and CDC-bound heterodimers with the closest mean temperature factors revealed differences in ligand interactions (Fig. 2*C* and Table S1). Specifically, the coordinated water molecule between H50, Y151, and the R2-OH of CDC increases the distance between the H50 and Y151 side chains, as compared with the TDC-bound structure, where they form a hydrogen bond. The key Fα S123 side chain makes a hydrogen bond with the R1-OH of TDC, which is lacking in CDC (Fig. 2*C*). Finally, the Y81-OH group shifts between 0.8 and 2.3 Å in the CDC-bound structure so that the aromatic side chain forms van der Waals contacts with both the five-membered ring and the carbon side chain from CDC (Fig. 2*C*).

**Figure 4:**
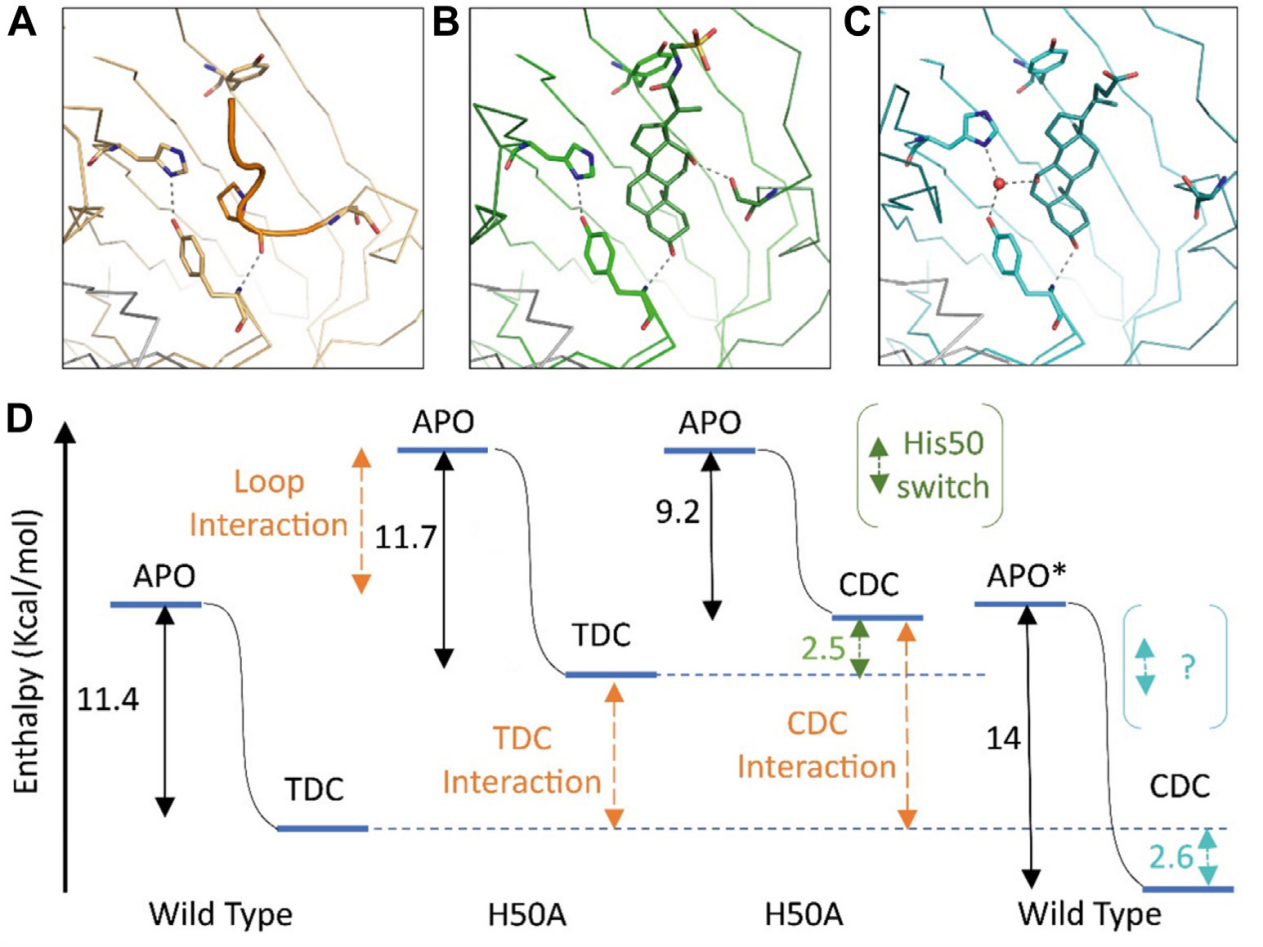
**Entropic contribution to specificity binding switch.***A*, Apo VtrA–VtrC structure (*orange ribbon*), with labeled key binding pocket residues (*stick*). Hydrogen bonds are indicated by *gray dotted lines*. The bile acid binding pocket is covered by the Fα extended loop (in *orange tube*), with the ring of P121 replacing the middle 6-member ring of the bile acid steroid backbone and the P121 backbone OH replacing the bile acid 3α-OH. H50 and Y181 form a similar hydrogen bond as seen in (*B*) the structure (*green ribbon*) bound to TDC (*dark green stick*). The R1-OH of TDC forms a hydrogen bond with Ser123 in the Fα helix (*dark green ribbon*). *C*, H50 conformation switch in structure (*cyan ribbon*) bound to CDC (*teal stick*) establishes a hydrogen bonding network to the R2-OH of CDC through an ordered water (*red sphere*). *D*, enthalpy coordinates for WT and H50A (labeled below) binding. Reaction from apo (*left bar*) to bound (TDC/CDC labeled bars) has a similar change for WT and H50A binding to TDC (*left black arrows*), suggesting a similar shift of both reactants and products to higher enthalpic energy for the mutation and a similar enthalpic energy for binding the Fα extended loop (*orange arrow*) and the TDC (*orange arrow*). The change in enthalpy for CDC binding to WT is larger. Assuming the free TDC and CDC are similar in the Apo state (∗), the WT protein bound to CDC reflects an enthalpic stabilization. CDC, chenodeoxycholate; TDC, taurodeoxycholate.

The residues from the Fα helix are variable in a superposition of the four heterodimers in the asymmetric unit of the CDC-bound crystal (Figs. 3*A* and S2*A*). The position of the Fα helix containing residue S123 is impacted by crystal packing and shifts away from the CDC binding site in one heterodimer compared with the others in the asymmetric unit (Figs. 3*A* and S2*A*). However, the different conformations of the Fα helix suggest this region can be mobile. In the TDC-bound crystal, S123 forms a hydrogen bond with TDC in two of the three heterodimers in the asymmetric unit (Fig. 3*B*). In the other heterodimer (chains C/D), part of the Fα helix containing S123 and an unstructured loop are not modeled because of poor electron density (7). However, the *B*-factors of the Fα helix in the three TDC-bound heterodimers trend lower than those in the four CDC-bound heterodimers (Fig. S3). This trend suggests that the hydrogen bond between TDC and S123 constrains the Fα helix near the binding pocket. Meanwhile, the lack of a hydrogen bond between CDC and S123 allows mobility in the Fα helix and alternates conformations dictated by the crystal contacts. In addition, S123 does not form any polar contacts in the apo VtrA–VtrC structure (7), because the Fα helix is shorter and the flexible loop preceding S123 is not ordered (Fig. 3*C*). This flexible loop forms contacts with the backbone of Y151 (Fig. 4*C*) and covers the biding pocket in the absence of any bile acid.

The positions of two loops surrounding the bile acid binding site of the VtrA–VtrC β-barrel near Y81 (VtrC residues 70–79) and near the interface with VtrA (VtrC residues 43–48) also differed in the CDC- and TDC-bound structures (Figs. 2*C* and S1). These loops adopt different conformations in the two crystals because of different interactions with other heterodimers in the asymmetric unit and with lattice contacts. The interface proximal loop containing VtrC residues 43 to 48 adopts distinct positions in the CDC- and TDC-bound structures (Fig. S2*C*). In the CDC-bound structure, this loop maintains a similar orientation in all four heterodimers. None of the side-chain residues from the CDC-bound loop make polar contacts with VtrA. In the TDC-bound structure, this loop rotates in all three heterodimers such that VtrC D45 moves toward a flexible loop in VtrA (residues 234–239). In one of the TDC-bound heterodimers, VtrC D45 forms a hydrogen bond with the backbone amide of VtrA E235 (Fig. S2*C*). Given the proximity to VtrA and the consistently alternate conformations displayed by all CDC-bound and TDC-bound heterodimers, the interaction between this loop and VtrA may be important for TDC-induced activation of *VtrB* expression. However, another explanation for these alternate conformations is differing pH in the solvents used to crystallize the CDC-bound (pH = 7.0) and TDC-bound (pH = 4.6) proteins (see the Experimental procedures section, (7)).

### Pocket residues in VtrC dictate its binding to bile acids

To test the contribution of residues in the bile acid binding pocket to TDC and CDC binding, we made mutant constructs of the VtrA–VtrC periplasmic domains so that their binding to bile acids could be compared with WT binding (Figs. 1*B* and S4). The mutants contain single amino acid substitutions of residues H50, Y81, S123, and Y151 with alanine or phenylalanine (Table 1). All VtrA–VtrC mutant constructs formed stable complexes in solution (Fig. S4). We then tested the binding of TDC and CDC to these VtrA–VtrC mutants. Most of the VtrA–VtrC mutants bound both TDC and CDC less favorably compared with WT, as shown by twofold (H50A) to 19-fold (Y151A) increases in *K*_*D*_ for TDC and twofold (Y81A) to sevenfold (Y151A) increases in *K*_*D*_ for CDC (Table 1). The exception to this trend was the S123A mutant, which had a similar *K*_*D*_ for both TDC and CDC compared with WT (Table S2).

Using the ITC technique to measure bile acid binding is advantageous in that it provides thermodynamic parameters of entropy (ΔS) and enthalpy (ΔH) in addition to binding constants. Considering the values of these parameters in light of the apo- and bile acid–bound structures can provide insight into how binding is mediated by interactions (from enthalpy, ΔH) and disorder (from entropy, TΔS). The enthalpy change of WT binding to TDC (ΔH = −11.4 kcal/mol) is less favorable than its binding to CDC (ΔH = −14.0 kcal/mol), whereas the entropic contribution of binding TDC (TΔS = −2.2 kcal/mol) is more favorable than binding CDC (TΔS = −5.2 kcal/mol) (Fig. 4). Consistent with these entropic values, a mobile loop from the apo structure (Fig. 4*A*, *orange tube*) is ordered upon bile acid binding. The more restricted freedom resulting from the WT VtrA–VtrC binding to CDC cannot be explained by the structure *B*-factors, suggesting the difference arises from additional ordering of solvent in the CDC-bound state. Accordingly, the CDC-binding specificity results from an ordered water interaction with the R2-OH (Fig. 4*C*). Additional contributions to the entropic difference could include the increased flexibility of the bound TDC with its longer R3-7Tail side chain (Table 2) conjugated to taurine with respect to the shorter unconjugated R3-5Tail side chain (Table 2) for CDC or from differential higher order protein interactions in the CDC- and TDC-bound states.

**Table 2:** Thermodynamic parameters of VtrA–VtrC binding with various bile acids determined by ITC

Bile acid	Activation	ΔG (kcal/mol)	ΔH (kcal/mol)	ΔS (cal/mol K)^a^	TΔS (kcal/mol)	*K*_*D*_ (nM)	R1^a^	R2^a^	R3^a^
TDC	+++	−9.2	−11.4	−7.4	−2.2	129.4	OH	H	7Tail
DC	+	−9.0	−13.3	−14.2	−4.2	232.0	OH	H	5Tail
GCDC	+	−9.1	−13.2	−13.6	−4.1	211.4	H	OH	7Tail
CA	−	−8.8	−13.3	−15.2	−4.5	377.1	OH	OH	5Tail
CDC	−	−8.7	−14.0	−17.8	−5.2	310.3	H	OH	5Tail

a As indicated in Fig. S1*A*, 5Tail and 7Tail refer to the number of carbon atoms (5 and 7) in the side chain (Tail) of each bile acid.

In addition to the thermodynamic differences in WT bile acid binding, the VtrC mutations had varying effects on the enthalpic and entropic contributions to binding. The Y81A mutation decreased the enthalpy change for both TDC and CDC binding when compared with WT as well as favorably altered the entropic contributions to positive values (Table 1). The difference in ΔH resulting from the Y81A mutation could result from a decrease in van der Waals contacts between either of the bile acids and the smaller alanine side-chain mutation compared with that with the WT tyrosine 81 side chain (Fig. 2). The increased entropy of the Y81A mutation suggests that the Y81 side chain promotes the stability of the bound state of the protein when compared with the free state, possibly through bile acid–induced organization of the mobile loop (Table 1). The entropy difference observed for TDC binding with respect to CDC binding to the Y81A mutant (Table 1) may reflect the observed ordering of Fα helix by S123 upon binding TDC (Figs. 2*B* and S3).

### A conformational switch in the binding pocket accommodates CDC

Mutation of H50 and Y151, whose side chains form a hydrogen bond in the WT TDC-bound structure and line the binding pocket (Figs. 2*B* and 4*B*), should theoretically decrease the enthalpy of binding to the bile acids. However, both H50A and Y151A had similar ΔH for TDC binding as the WT protein (Table 1). The apo structure of VtrA–VtrC experiences a conformational change, where an extended loop from the Fα helix covers the bile acid–binding site. In the apo structure, the H50–Y151 pair forms the same hydrogen bond as in the TDC-bound state, but they instead interact with P121 from this loop. The hydrophobic ring of P121 is replaced by the middle six-membered ring from the TDC steroid nucleus upon binding (Fig. 4*A*). Thus, the similar ΔH values for the mutant and WT proteins likely reflect a similar entropic contribution to binding the Fα helix loop in the apo state as to binding the steroid nucleus in the TDC-bound state. The enthalpic energies for each state (apo and TDC bound) in the H50A mutant should increase to the same extent (Fig. 4*D*). Mutation of the H50–Y151 hydrophobic anchor for the Fα extended loop should also increase its flexibility. The elevated changes in entropy for TDC binding to the H50A and Y151A mutants support this notion (Table 1).

The Y151A mutant also displayed similar ΔH as WT when binding CDC, but the ΔH value for the H50A mutant was decreased (Table 1). In the CDC-bound structure, the pocket changes to accommodate the R2-OH of CDC. The position of the H50 side chain switches to form a hydrogen bond with an ordered water instead of the Y151 side chain OH. The ordered water establishes a hydrogen bond network between H50, Y151, and the R2-OH from CDC (Figs. 2*A* and 4*C*). The H50 conformational switch accommodates CDC binding, which is reflected in a decreased ΔH of the H50A mutation for binding CDC with respect to WT. For the H50A mutant, the change in enthalpy for CDC binding is smaller than that of TDC binding, resulting in an increased enthalpic energy that might reflect the loss of interaction by the conformation switch (Fig. 4*D*).

The Y151F mutation distinguishes the ability of the side chain to form hydrogen bonds from its aromatic contribution to hydrophobic interactions. The Y151F mutation did not alter the ΔH of TDC binding much compared with WT. On the other hand, the Y151F mutation decreased the enthalpic contribution of CDC binding compared with WT (Table 1). This decrease would be consistent with the loss of the Y151 hydrogen bond, destabilizing the CDC-bound state where the hydrogen bonding network accommodates the R2-OH (Fig. 2*A*), and mirrors the decreased ΔH of the H50A mutation. In addition, the ΔH and TΔS values of bile acid binding to WT and mutant VtrC follow an opposing linear trend (Fig. S5). This trend suggests that the VtrC point mutations resulted in entropy–enthalpy compensation for ligand binding, as seen in many interactions between small molecules and macromolecules (12).

### Other bile acids show similar trends in binding thermodynamics

To test this model of ligand-specific activation mediated by the conformation of H50, the binding affinities of additional bile acids to WT VtrA–VtrC were measured by ITC (Tables 2 and S3). Among the bile acids found in humans, TDC is a high inducer of *VtrB* transcription, whereas CDC is a noninducer (6). Two intermediate inducers, deoxycholate (DC) and glycochenodeoxycholate (GCDC), and one additional noninducer (6), cholate (CA), were tested using ITC.

The intermediate-inducer GCDC bound to VtrA–VtrC with a binding affinity of 211.4 nM (Table 2), similar to that of TDC but not CDC (Table S3). However, the ΔH of GCDC binding (−13.2 kcal/mol) was closer to that of CDC (−14.0 kcal/mol) and more negative than that of TDC (−11.4 kcal/mol) (Table 2). The increased enthalpic stability of GCDC and CDC binding can be explained by the R2-OH present in both bile acids, which forms a hydrogen bonding network that orients H50 further away from Y151 in the CDC-bound crystal structure (Fig. 2*A*). The higher binding affinity of GCDC compared with that of CDC can be explained by the higher entropy of GCDC binding (TΔS = −4.1 kcal/mol) compared with CDC binding (TΔS = −5.2 kcal/mol) (Table 2). Accordingly, GCDC has an R3-7Tail that is more flexible than the R3-5Tail of CDC (Table 2).

Intermediate-inducer DC bound to VtrA–VtrC with a binding affinity of 232.0 nM, which is similar to that of TDC and GCDC (Table 2). DC had a lower entropy (TΔS = −4.2 kcal/mol) compared with TDC (TΔS = −2.2 kcal/mol) (Table 2). This lower entropy reflects the shorter R3-5Tail of DC compared with the R3-7Tail of TDC and follows the same trend in entropy as CDC and GCDC. However, DC had a more negative ΔH of −13.3 kcal/mol compared with that of TDC (Table 2). The reason for this increased enthalpic stability for DC binding is not clear, as DC and TDC both have an R1-OH (Table 2), but may reflect the shorter R3-5Tail of DC and other changes in binding compared with TDC.

The noninducer CA had a binding affinity of 377.1 nM, similar to that of CDC (Table 2). The ΔH of CA binding was −13.3 kcal/mol, which is closer to the ΔH of −14.0 kcal/mol for CDC binding (Table 2). Like CDC, CA has an R2-OH that can potentially form a similar hydrogen bonding network seen in the CDC-bound crystal structure. CA also has an R1-OH like TDC (Table 2). Yet CA has a lower TΔS = −4.5 kcal/mol compared with the TΔS = −2.2 kcal/mol of TDC. This lower entropy of CA binding correlates with the shorter R3-5Tail of CA compared with the R3-7Tail of TDC (Table 2).

Comparing the thermodynamic parameters for the binding of these five bile acids reveals two general trends. Bile acids with an R2-OH have more negative ΔH values compared with those with an R1-OH, except in the case of DC. Meanwhile, bile acids with an R3-5Tail have lower TΔS values compared with those with an R3-7Tail. These trends support the model of ligand-specific activation of VtrA–VtrC because of molecular differences between bile acids.

### Residue S123 dictates bile acid specificity for inducing *VtrB* transcription

**Figure 5:**
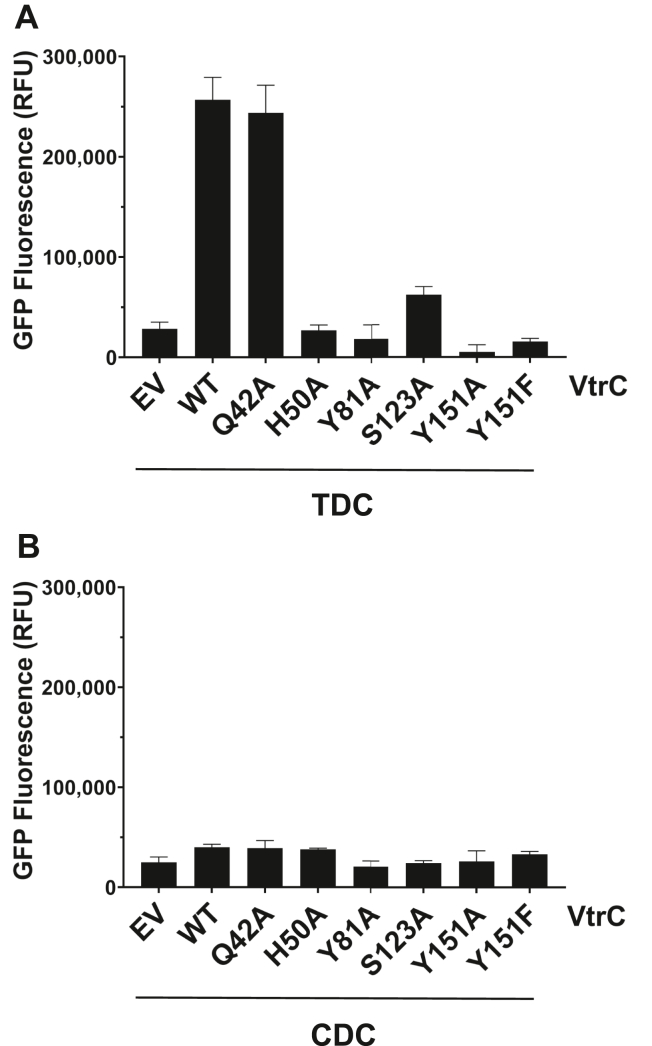
**Mutation of residues involved in bile acid binding disrupts TDC-induced signaling, whereas CDC does not induce signaling.***A*, GFP fluorescence from a P_*vtrB*_*-gfp* transcriptional reporter in *Vibrio parahaemolyticus* POR1Δ*vtrC* strains expressing FLAG-VtrC (WT) and single amino acid substitution mutants upon treatment with TDC. EV, empty vector control. *B*, GFP fluorescence of POR1Δ*vtrC* strains in (*A*) treated with CDC. Results are the means of three technical replicates. Results are representative of three biological replicates. Error bars denote SD. ∗*p* < 0.05; ∗∗∗*p* < 0.0001; comparisons to the WT strain determined by one-way ANOVA followed by Tukey’s multiple comparison test. CDC, chenodeoxycholate; TDC, taurodeoxycholate.

The S123A mutation did not change the binding affinity of VtrA–VtrC to either bile acid (Table 1). However, the ΔH of TDC binding decreased and TΔS shifted to a positive value, likely because of the loss of a stabilizing hydrogen bond between the TDC R1-OH and S123 on the Fα helix (Figs. 2*B* and 4*B*). Alternately, the ΔH and TΔS of CDC binding was similar to that of the WT protein because S123 does not form a hydrogen bond with CDC (Figs. 2*A* and 4*C*). Because binding to bile acids does not change for the S123A mutant, we suspected that the conformation of the Fα helix imposed by the S123 side chain might dictate the specificity of the *VtrB* transcription factor activation. To test this hypothesis, we used a GFP reporter assay in which a *V. parahaemolyticus* POR1*ΔvtrC* strain (see the Experimental procedures section) expressed N-terminally FLAG-tagged *vtrC* (WT) or single amino acid substitutions of *vtrC* (including Q42A, H50A, Y81A, S123A, Y151A, and Y151F) from a plasmid under the control of an arabinose-inducible promoter. The strains also contained a second plasmid with the *gfp* gene under the control of the *V. parahaemolyticus VtrB* promoter (8) with the 300 bp upstream region of the *VtrB* open reading frame (P_*VtrB*_*-gfp*). The strains were treated with either 100 μM TDC or 100 μM CDC, and GFP fluorescence intensity (FI) and absorbance at 600 nm were measured every 5 min for 1 h. The GFP fluorescence readings were then normalized by absorbance at 600 nm, and the fluorescence readings at the 1 h time point are plotted in Figure 5.

Substitution of S123 with alanine resulted in a significant fourfold decrease in GFP fluorescence in response to TDC compared with the WT strain, suggesting this residue plays a role in transcription factor activation in response to binding TDC. In the presence of CDC, the S123 mutant exhibited similar low levels of GFP fluorescence that was not significantly different than the empty vector. The WT-level binding of the S123A mutant to both TDC and CDC, together with the selective activation of *VtrB* transcription in response to TDC, but not CDC, supports a role for the S123 hydrogen bond stabilization of the Fα helix in TDC-specific activation of the VtrA–VtrC transcription factor.

### Bile acid–binding residues induce *VtrB* transcription

Substitution of the remaining binding residues H50, Y81, and Y151 with alanine or phenylalanine all resulted in greater than 75% decrease in GFP fluorescence in response to TDC compared with the WT strain (Fig. 5*A*). Meanwhile, the Q42A mutation, located outside the binding pocket (7), had similar GFP fluorescence as the WT strain. WT VtrC and all VtrC point mutants were expressed at similar levels in the uninduced and TDC-induced states (Fig. S6). The Q42A mutant was previously shown to express similar levels of protein compared with WT VtrC (7). Similar to the results for S123A, treatment of the WT and mutant strains with CDC induced GFP fluorescence at low levels that were not significantly different from an empty vector control (Fig. 5*B*). These results indicate that the tested binding pocket residues are all important for activation of *VtrB* transcription in response to TDC. Meanwhile, CDC does not induce transcription of *VtrB* in any of the WT or mutant strains.

As shown in Figure 1, the bile acids TDC and CDC can compete to induce or suppress *VtrB* transcription, respectively. Primary bile acids such as CDC and CA are made in the liver and conjugated to taurine or glycine prior to secretion into the duodenum (11). Bacteria in the intestines can extensively modify these bile acids using reactions such as deconjugation by bile salt hydrolases (BSHs), hydroxylation, or dehydroxylation to form unconjugated primary bile acids and secondary bile acids like DC (11). Primary and secondary bile acids are reabsorbed from the intestines and reconjugated in the liver (11, 13), producing conjugated secondary bile acids like TDC.

Conjugated forms of the secondary bile acids, such as TDC, were previously observed as high inducers of *VtrB* transcription, whereas the unconjugated primary bile acids CDC and CA do not induce transcription (6). Meanwhile, conjugated primary bile acids and unconjugated DC induce intermediate levels of transcription. Despite these observations, the mechanism of *VtrB* activation remained elusive until the discovery of VtrA–VtrC, now known as a co-component signal transduction system that responds to the bile acid TDC and activates *VtrB* (4, 7). The high-inducing bile acids such as TDC have an R1-OH and an amino acid (taurine or glycine) conjugated to the end of the R3-7Tail side chain (Fig. 1*A* and Table 2). The noninducing bile acids have an R2-OH instead of R1-OH group (or both for CA) and no conjugated amino acids on the R3-5Tail side chain (Table 2). The intermediate-inducing bile acids have just one of either the R1-OH or a conjugated R3-7Tail side chain (Table 2).

In this study, we discover the differential interaction between noninducing bile acids and VtrA–VtrC. The noninducing CDC lacks an R1-OH and does not interact with S123, a key residue for transcription activation upon binding the R1-OH of TDC (Fig. 2*B* and Table 2). Instead, CDC has an R2-OH that causes a conformational switch in the VtrC H50 side chain (Figs. 2*A* and 4*C*). This conformational switch disrupts the H50–Y151 interaction that is also important for activating transcription. Therefore, the position of hydroxyl groups in the steroid nucleus and the presence of an amino acid conjugated to the carbon side chain are correlated with the ability of a bile acid to induce *VtrB* transcription *via* VtrA–VtrC.

Modifications of bile acids by BSHs have various proposed benefits for gut bacteria, including providing a nutrient source through release of conjugated amino acids, strengthening the bacterial membrane through incorporation of bile acids and cholesterol, or reducing the acidic and detergent properties of bile acids that are toxic to bacteria (11, 14). Recent studies have also shown that the modification of bile acids by BSHs of different microbiome species can provide protection against colonization by enteric pathogens such as *V. cholerae* and *C. difficile* by altering the makeup of the bile acid pool in the intestines (15, 16). In the case of *V. parahaemolyticus*, higher levels of BSH activity in the intestines may produce a greater proportion of unconjugated primary bile acids like CDC that suppress T3SS2 expression. Although beyond the scope of this study, we predict that gut microbiota BSH activity and the makeup of the intestinal bile acid pool may also be important for determining whether *V. parahaemolyticus* passes through the host intestines as a friend or a foe.

### Residue H50 is important for *VtrB* transcription under acidic and neutral conditions

*V. parahaemolyticus* encounters greatly different pH environments as the bacterium travels through the human gastrointestinal tract after being consumed. The human gastrointestinal tract varies from pH 1.0 to 2.0 in the stomach to pH 6.6 to 7.5 in the small intestines and pH 6.5 to 7.0 in the large intestines (17). Since histidine residues can have different protonation states under various physiologic pH conditions, we tested whether pH alters the activity of VtrA–VtrC in response to TDC.

**Figure 6:**
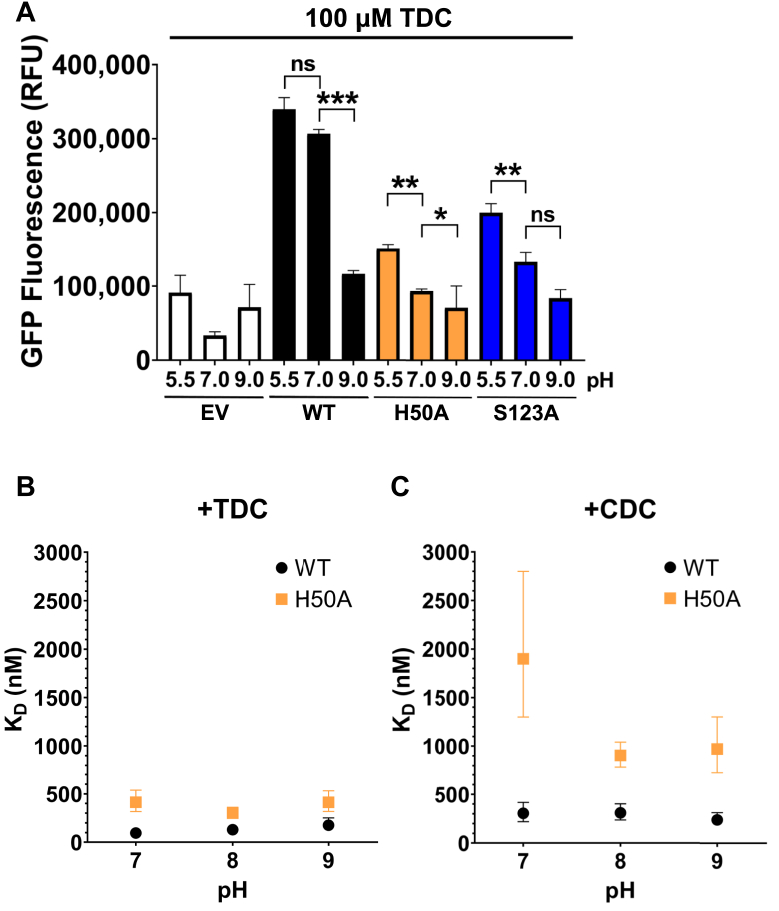
**H50A and S123A mutations reduce TDC-induced transcription under acidic and neutral conditions.***A*, GFP fluorescence from a P_*vtrB*_*-gfp* transcriptional reporter in *Vibrio parahaemolyticus ΔvtrC* strains expressing VtrC (WT) and single amino acid substitution mutants upon treatment with 100 μM TDC under different pH conditions. Results are the mean of three technical replicates. Error bars denote SD. ns, nonsignificant; ∗*p* < 0.05; ∗∗*p* < 0.005; ∗∗∗*p* < 0.0001; determined by one-way ANOVA followed by Tukey’s multiple comparison test. *B*, *K*_*D*_ of TDC binding WT and H50A protein constructs under various pH conditions measured by ITC. Error bars denote 68.3% confidence intervals. *C*, *K*_*D*_ of CDC binding WT and H50A protein constructs under various pH conditions measured by ITC. Error bars denote 68.3% confidence intervals. ITC, isothermal titration calorimetry; TDC, taurodeoxycholate.

We cultured the WT, H50A, and S123A strains and performed the GFP reporter assay in media adjusted to pH 5.5 using hydrochloric acid, pH 7.0 (no adjustment), and pH 9.0 using sodium hydroxide. For the WT strain, the level of GFP fluorescence at pH 5.5 and 7.0 was not significantly different, whereas it decreased at pH 9.0 (Fig. 6). For the H50A strain, the GFP fluorescence was lower than that of the WT strain under each pH condition. However, the decrease in GFP fluorescence shifted to a lower pH (between pH 5.5 and 7.0). The fluorescence at pH 7.0 was not significantly different from the fluorescence at pH 9.0. A similar trend was seen for the S123A mutant, except the fluorescence at pH 7.0 was significantly higher than at pH 9.0. These results indicate H50 and S123 are important for activating *VtrB* transcription under acidic and neutral conditions.

We reasoned that the different levels of *VtrB* transcription under different pH conditions may be a result of pH-dependent changes in the binding affinity of TDC and CDC. To test the effect of pH on binding affinity, we performed additional ITC experiments with the VtrA–VtrC periplasmic domains. The ITC results in Table 1 were measured using a pH 8.0 buffer (see the Experimental procedures section). We then purified the WT and H50A constructs of the VtrA–VtrC periplasmic domains in pH 7.0 and 9.0 buffers. The binding affinities of TDC and CDC to each protein construct were measured using these same buffers at pH 7.0 and 9.0 (Table S4). The *K*_*D*_ measurements *versus* pH for TDC and CDC are plotted in Figure 6, *B* and *C*, respectively. The WT protein bound TDC with slightly higher *K*_*D*_ with increasing pH (Fig. 6*B*). However, these *K*_*D*_ values were all within the 68.3% confidence intervals (CIs) of each other and were not significantly different (Table S4). Similarly, the WT protein bound CDC with similar affinities under each pH condition (Fig. 6*C*). Meanwhile, the H50A construct bound TDC with similar affinities under each pH condition, with higher *K*_*D*_ values compared with that of WT with TDC (Fig. 6*B*). Interestingly, the H50A construct bound CDC with a higher *K*_*D*_ around 1900 nM at pH 7.0 compared with *K*_*D*_ values of around 900 nM under pH 8.0 and 9.0 conditions (Fig. 6*C*). However, the lower limit of the 68.3% CI of the *K*_*D*_ at pH 7.0 was close to the upper limit of the corresponding CI at pH 9.0 (Table S4). As a result, we cannot confidently say the binding affinity of H50A with CDC is significantly different at pH 7.0 compared with pH 8.0 and 9.0.

These ITC results indicate that the bile acid binding affinities of the WT and H50A constructs do not change significantly under different pH conditions. The different levels of *VtrB* transcription under various pH conditions (Fig. 6*A*) are not a result of changes in bile acid binding. Instead, residues outside the bile acid binding site may control the differential activity of the complete VtrA–VtrC protein in response to pH.

The model p*K*a of the histidine side chain, which is the p*K*a value of histidine in water, is well known and measured to be 6.50 (18). However, the p*K*a of ionizable residues in a protein can vary because of desolvation effects from the burial of the residue in the protein and intraprotein interactions with nearby residues (18). The empirical p*K*a predictor PROPKA 3.4.0 (https://www.ddl.unimi.it/vegaol/propka.htm) (19, 20) suggests this to be the case for the H50 switch residue. The predicted p*K*a of H50 was 3.75 in the apo structure, 3.68 in the TDC-bound structure, and 5.44 in the CDC-bound structure. The apo structure was crystallized in pH 5.6 buffer, the TDC-bound structure in pH 4.6 buffer (7), and the CDC-bound structure in pH 7.0 buffer (see the Experimental procedures section).

**Figure 7:**
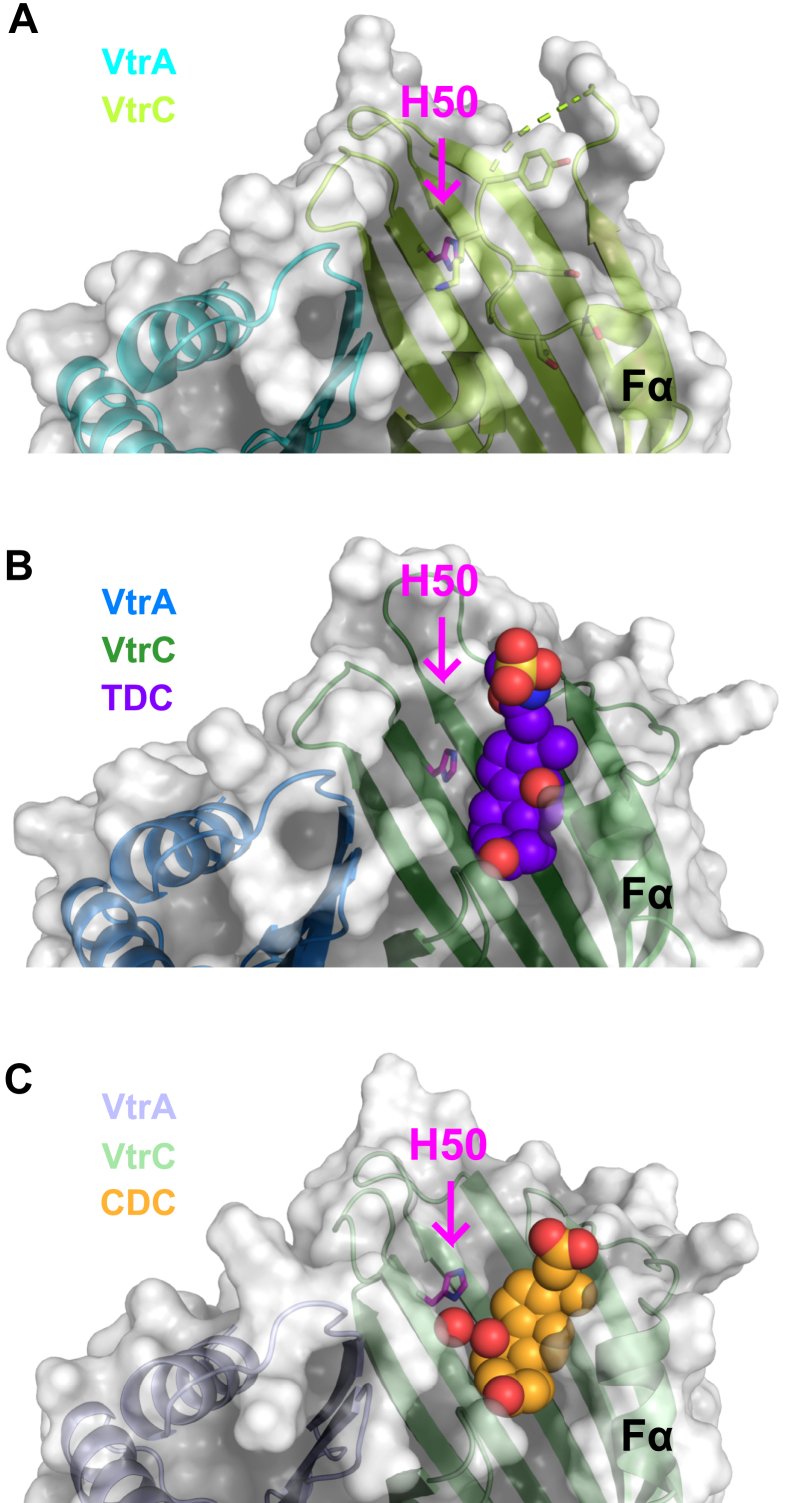
**Burial of VtrC H50 in the apo, TDC-, and CDC-bound structures.***A*, overlay of *surface and ribbon models* of apo VtrA–VtrC. Side chains of H50 and residues in the flexible loop covering binding pocket are shown as *sticks*. H50 is indicated in *magenta*. *B*, overlay of *surface and ribbon models* of TDC-bound VtrA–VtrC. TDC is shown as *spheres*. Side chain of H50 is shown as *sticks* and indicated in *magenta*. *C*, overlay of *surface and ribbon models* of CDC-bound VtrA–VtrC. CDC and coordinated water molecule are shown as *spheres*. Side chain of H50 is shown as *sticks* and indicated in *magenta*. CDC, chenodeoxycholate; TDC, taurodeoxycholate.

To predict the p*K*a shift caused by the desolvation effect, PROPKA 3.4.0 calculates how buried a residue is in the protein structure. The desolvation effect raises the p*K*a of buried acidic residues and lowers the p*K*a of buried basic residues (19). According to PROPKA 3.4.0, VtrC H50 was 77% buried in the apo structure, 75% buried in the TDC-bound structure, and 63% buried in the CDC-bound structure. These percent burial values correspond with the burial of H50 seen in the crystal structures. In the apo structure, H50 is covered by the flexible loop containing the short Fα helix (Fig. 7*A*). In the TDC-bound structure, this loop is displaced, but H50 is covered by TDC binding (Fig. 7*B*). In the CDC-bound structure, the position of H50 is shifted slightly away from Y151 because of the interaction with a coordinated water molecule (Fig. 2*C*) and H50 is more exposed (Fig. 7*C*).

Based on the similar predicted p*K*a values and percentage burial of H50 in the apo- and TDC-bound structures, we speculate that the H50–Y151 interaction (Fig. 2*B*) primes VtrA–VtrC for activation. Upon displacement of the flexible loop by TDC binding and formation of a hydrogen bond between TDC and S123, VtrA–VtrC is activated. However, when CDC binds to VtrA–VtrC, the protein complex is no longer primed for activation because the position of H50 is shifted upward by the presence of a coordinated water molecule between H50 and Y151 (Fig. 2*A*).

However, the H50 p*K*a predictions do not provide an explanation for why VtrA–VtrC induces transcription upon binding TDC under pH 5.5 and 7.0 conditions but not under pH 9.0 conditions (Fig. 6). The predicted p*K*a of 3.7 for H50 in the TDC-bound structure is lower than the three pH conditions we tested. Based on this predicted p*K*a, H50 would be protonated in less than 50% of VtrA–VtrC dimers at pH 5.5. At pH 7.0 and 9.0, gradually lower proportions of VtrA–VtrC would have protonated H50 residues. These values are more consistent with the pH titration of TDC binding measured by ITC for the periplasmic region (Fig. 6*B*). Of note, the structures used in the PROPKA predictions contained only the periplasmic domains of VtrA–VtrC. Since there are currently no structures of full-length VtrA–VtrC, the effect of different pH conditions on the whole protein complex is not clear.

A previous study showed that expression of truncated VtrA with the cytosolic DBD alone was not sufficient to activate *VtrB* transcription, whereas a construct containing the putative VtrA transmembrane domain and the DBD was sufficient (8). These results suggest that full-length VtrA–VtrC may form an inactive dimer of dimers in the apo state. Upon TDC binding, this dimer of dimers may change conformation to activate the DBD. Evidence for a dimer of dimers can be seen in the TDC-bound crystal of the VtrA–VtrC periplasmic domains (7). This crystal contains dimer–dimer lattice contacts, and analysis of higher order interfaces in the TDC-bound crystal (PDB ID: 5KEW) (7) with PDBePISA (http://www.ebi.ac.uk/pdbe/pisa/pistart.html) (21) indicated that this dimer–dimer is stable in solution. However, PDBePISA analysis (21) of the apo- (PDB ID: 5KEV) (7) and CDC-bound crystals indicated that the VtrA–VtrC periplasmic heterodimer was the only stable quaternary structure in solution for both crystals. The formation of a dimer of dimers in the periplasmic domains upon TDC binding may cause a conformation change in the apo dimer to activate the DBD.

## Conclusions

**Figure 8:**
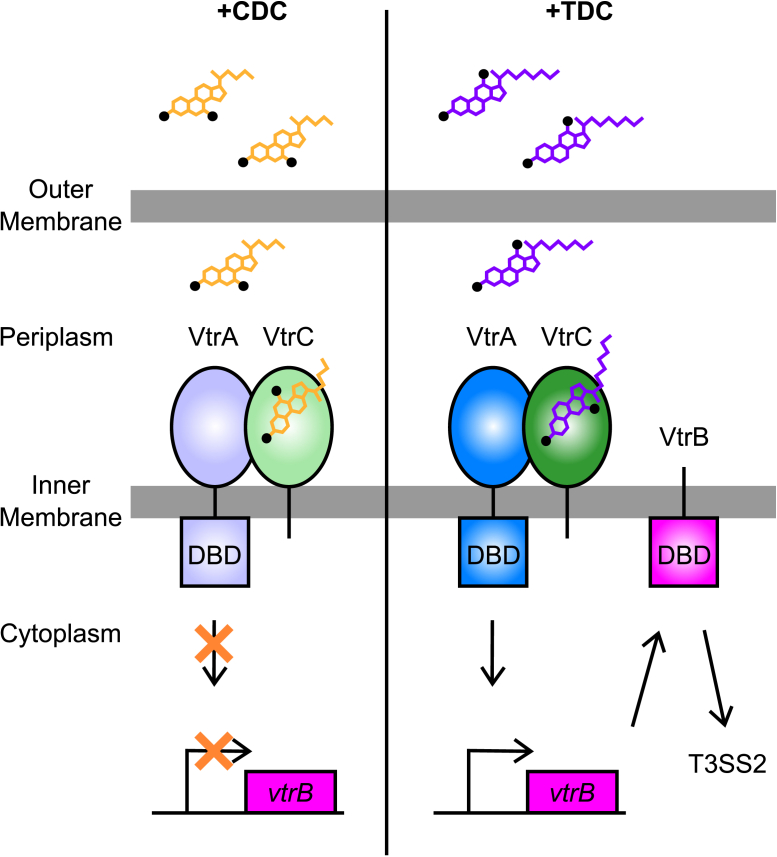
**CDC and TDC binding to VtrA–VtrC produce different outcomes on *VtrB* transcription.***Left*, when the bile acid CDC binds to the periplasmic domain of VtrC, the cytoplasmic DNA-binding domain (DBD) of VtrA does not induce transcription of *VtrB*. *Right*, in contrast, when TDC binds to the periplasmic domain of VtrC, the VtrA DBD induces transcription of *VtrB*, leading to the expression of another transmembrane protein containing a cytoplasmic DBD, VtrB. Subsequently, VtrB induces expression of type III secretion system 2 (T3SS2) encoding genes. Hydroxyl groups attached at different positions on CDC and TDC are depicted as *black dots*. CDC, chenodeoxycholate; TDC, taurodeoxycholate.

Herein, we demonstrate the molecular determinants of TDC and CDC that dictate whether the co-component signaling system VtrA–VtrC can transcriptionally activate *VtrB* and the pathogenic T3SS2 (Fig. 8). CDC and TDC bind to the same hydrophobic pocket in the VtrA–VtrC periplasmic domain complex but form different interactions with binding pocket residues. Functional and binding assays of various VtrA–VtrC mutants showed that the hydrogen bonds between VtrC H50 and Y151 and the TDC R1-OH and S123 are important for activating *VtrB* transcription (Figs. 2*B* and 5*A*). The van der Waals interaction between TDC and Y81 is also important for indiscriminate binding of the bile acids, and its absence suppresses transcription. In the case of CDC binding, the H50 side chain switches conformation to allow hydrogen bonding to an ordered water molecule in between these two residues and the R2-OH specific to CDC (Figs. 2*A* and 4*C*). There is no interaction between S123 and CDC because CDC has an R2-OH instead of an R1-OH (Figs. 1*A* and 2*A*). The ability of S123 to stabilize the Fα helix and extended loop when bound to TDC but not CDC might explain the specificity between the two bile acids for activating *VtrB* transcription (Fig. S3). Mutation of this residue does not alter binding to TDC but instead increases the entropy (Table 1) and reduces the ability of VtrA–VtrC to activate transcription of *VtrB* (Fig. 5*A*). Alternately, the TΔS of the S123A mutant does not change much for CDC, which does not activate transcription. The VtrA–VtrC transcription factor might harness the lower entropy of the S123-stabilized Fα helix in the TDC-bound state to dimerize for transcription activation. The structure determination and ITC binding experiments in this study are limited to the periplasmic domains of VtrA–VtrC and pH response of binding to the periplasmic region compared with transcription activation by the full-length VtrA–VtrC differs. More studies are needed to determine the molecular mechanism of the inducing activity of TDC and noninducing activity of CDC in the context of full-length VtrA–VtrC.

## Experimental procedures

### Bacterial strains and cell culture

The *V. parahaemolyticus* POR1 *vtrB-3XFLAG* and POR1Δ*vtrC* strains were derived from POR1 (clinical isolate RIMD2210633 containing deletions of TDH toxins). The *vtrB-3XFLAG* strain contains an insertion of three tandem FLAG epitopes at the 3′ end of the native *VtrB* coding region, made using the Ori6K/SacB suicide vector pDM4 (22). The POR1Δ*vtrC* strain contains a deletion of the *vtrC* coding sequence (nucleotides 34–486) (7). Single amino acid substitution mutants of VtrC were generated using site-directed mutagenesis of the vectors pACYC-Duet-VtrC/VtrA and pBAD-FLAG-*vtrC* (7). Primers used for cloning are listed in Table S5. For vector-induced expression of FLAG-*vtrC* variants under the control of the arabinose-inducible promoter, pBAD-FLAG-*vtrC* plasmids were introduced into POR1Δ*vtrC* using standard triparental mating. Subsequently, a pRU1701 (23) derivative plasmid containing the 300 bp upstream of *vtrB* (pRU1701 *vtrB* −300 bp) was introduced into the POR1Δ*vtrC* pBAD-FLAG-*vtrC* strains using *E. coli* S17-1 (λ*pir*). The *V. parahaemolyticus* POR1 *vtrB-3XFLAG* strain was cultured in marine LB (MLB) broth (LB broth with 3% NaCl). Strains containing the pBAD and pRU1701 plasmids were cultured in MLB broth supplemented with 100 μg/ml gentamicin and 250 μg/ml kanamycin. *V. parahaemolyticus* cultures were grown at 30 °C or as otherwise indicated.

### Antibodies

FLAG antibody was purchased from Sigma–Aldrich (F3165).

### VtrB-3XFLAG expression assay

*V. parahaemolyticus* POR1 *vtrB-3XFLAG* strain was grown overnight in MLB medium at 30 °C. The day of the experiment, new cultures were inoculated with an absorbance of 0.3 at 600 nm in MLB using the overnight cultures and incubated at 30 °C for 2.5 h. Cultures were then induced with TDC and CDC at the indicated concentrations and incubated at 37 °C for 1 h. Bacterial cultures equivalent to an absorbance of 1.0 at 600 nm were collected, and cell pellets were resuspended in 2× protein sample buffer. Protein expression was detected by Western blot analysis.

### GFP reporter assay

*V. parahaemolyticus* POR1Δ*vtrC* pBAD-FLAG-*vtrC* and pRU1701 strains were grown in MLB medium at 30 °C overnight. The day of the experiment, new cultures were inoculated with an absorbance of 0.6 at 600 nm in MLB supplemented with 1% arabinose using the overnight cultures and incubated at 30 °C. After 2 h of incubation, samples were prepared in triplicate by diluting the previous cultures to an absorbance of 0.3 at 600 nm in MLB supplemented with 1% arabinose and either 100 μg/ml TDC or 100 μg/ml CDC. Samples (200 μl) were transferred to a 96-well plate and incubated at 37 °C in the plate reader. GFP FI was measured every 5 min using the BMG Labtech CLARIOstar^Plus^ plate reader with an excitation filter of 470 nm and emission filter of 515 nm. Absorbance at 600 nm was measured after each GFP FI measurement, and these values were used to normalize GFP FI to cell density. Plates were shaken in between each GFP FI and absorbance at 600 nm measurement.

### Protein expression and purification

The periplasmic domain of VtrC (amino acids 31–161) and mutants with single amino acid mutations were coexpressed with the periplasmic domain of VtrA (amino acids 161–253) using variants of the pACYC-Duet-VtrC/VtrA construct in which VtrC has an N-terminal hexahistidine tag (7). The constructs were expressed in *E. coli* BL21(DE3) cells and purified using nickel-affinity and size-exclusion chromatography (SEC) (7). Briefly, all cultures were grown in LB at 37 °C until an absorbance of 0.5 to 0.6 at nm and induced with 0.4 mM IPTG overnight at 16 °C. Cells were lysed in buffer A (50 mM Tris [pH 8.0] and 100 mM NaCl) supplemented with 1 mM phenylmethylsulfonyl fluoride (Sigma) using a cell disrupter (Emulsiflex C3; Avestin, Inc). Clarified and filtered (0.45 μm pore size) lysates were incubated with Qiagen Ni–nitilotriacetic acid resin (catalog no.: 30210) for 30 min at 4 °C with nutation. Lysate and resin were applied to a gravity column. Protein-bound resin was washed and eluted with buffer A supplemented with 15 mM (wash) and 250 mM (elution) imidazole, as described previously (7). Eluted proteins were further purified by SEC on a Superdex 200 Increase 10/300 GL column (Cytiva) with buffer A. For crystallographic studies, the VtrA–VtrC heterodimer bound to the bile acid CDC was purified by nickel-affinity chromatography with 0.5 mM CDC in all buffers. The final SEC purification was performed with buffer B (10 mM Tris [pH 8.0] and 10 mM NaCl) supplemented with 0.5 mM CDC. CDC-bound protein was buffer exchanged into buffer B without bile acid prior to crystallographic studies.

### Crystallization and X-ray data collection

Crystals of the VtrA–VtrC periplasmic domain heterodimer bound to the bile acid CDC were grown using the hanging-drop vapor diffusion method from drops containing 1 μl of protein (4.8 mg/ml) and 1 μl of reservoir solution (16% polyethylene glycol 3350, 0.2 M calcium chloride) and equilibrated over 250 μl of reservoir solution. Crystals typically appeared in 2 to 7 days at 20 °C and grew to their maximal extent by 1 week. Crystals were cryoprotected by transferring to a final solution of 18% polyethylene glycol 3350, 0.2 M calcium chloride, 10 mM Tris (pH 8.0) and 35% glycerol, and flash-cooled in liquid nitrogen.

Data were collected at APS beamline 19-ID at 100 K and were indexed, integrated, and scaled using the HKL-3000 program package (24). CDC-bound VtrA–VtrC crystals belonged to space group C2 with unit cell parameters of a = 142.60 Å, b = 41.76 Å, c = 168.96 Å, and β = 91.57° and contained four molecules each of VtrA–VtrC heterodimer per asymmetric unit, with a solvent content of 45%. Data collection statistics are provided in Table S6.

### Phase determination and structure refinement

Phases for the CDC-bound VtrA–VtrC heterodimer were obtained by the molecular replacement method in the program *Phaser* (https://www.ucl.ac.uk/∼rmhasek/phaser.html) (25) using the coordinates for the TDC-bound VtrA–VtrC heterodimer (PDB ID: 5KEW). Manual model building was performed in the program *Coot* (https://www2.mrc-lmb.cam.ac.uk/personal/pemsley/coot/) (26). Positional and isotropic ADP and TLS ADP refinement was performed to a resolution of 2.08 Å using the program Phenix (https://phenix-online.org/) (27) with a random 3.70% of all datasets aside for an *R*_free_ calculation. The current model contains four VtrA–VtrC heterodimers in the asymmetric unit, each bound to one molecule of CDC, as well as four calcium ions and 292 water molecules. A *Molprobity* (28) generated Ramachandran plot indicates that 96.4% of residues are in the most favored regions and 0.1% (one residue) is in disallowed regions. Phasing and model refinement statistics are provided in Table S6.

### ITC

All variants of the VtrA–VtrC periplasmic domain complex were dialyzed against the assay buffer (50 mM Tris [pH 8.0] and 100 mM NaCl) overnight at 4 °C. Solutions of individual bile acids (TDC, CDC, DC, GCDC, and CA) were each prepared by dissolving dry powder (Sigma) with the same dialysis buffer to a concentration of 200 μM. ITC experiments were performed at 25 °C on a MicroCal iTC200 system (Malvern), with reference power at 5 μcal/s and stirring rate at 750 rpm. Measurements were generally performed as 21 injections of 200 μM TDC or CDC (1 μl for the first injection and 2 μl for injections 2–21) into approximately 200 μl of 20 μM VtrA/C. ITC data were integrated and analyzed using NITPIC 1.3.0 (https://www.utsouthwestern.edu/research/core-facilities/mbr/software/) (29, 30) and SEDPHAT version 15.2b (https://sedfitsedphat.github.io/sedphat/default.htm) (31). ITC data plots were prepared with GUSSI 1.4.2 (https://www.utsouthwestern.edu/research/core-facilities/mbr/software/) (32).

For ITC experiments performed under pH 7.0 and 9.0 conditions, the VtrA–VtrC periplasmic domain complex was purified as described previously, except the final purification by SEC was performed using pH 7.0 (50 mM Tris [pH 7.0] and 100 mM NaCl) and pH 9.0 (50 mM Tris [pH 9.0] and 100 mM NaCl) buffers. Protein and bile acid samples for ITC were prepared as aforementioned using the pH 7.0 and 9.0 buffers.

## Data availability

Structure factors and coordinates for the VtrA–VtrC and CDC complex were deposited in the PDB under the accession code 8DML.

## Supporting information

This article contains supporting information (Tables S1, S2, S3, S4, S5, S6, Figs. S1, S2, S3, S4, S5, S6, S7) (33).

## Conflict of interest

The authors declare that they have no conflicts of interest with the contents of this article.
