# Supplementary material for: Molecular determinants for differential activation of the bile acid receptor from the pathogen Vibrio parahaemolyticus
Source: J Biol Chem. 2023 Mar 7;299(4):104591. doi: 10.1016/j.jbc.2023.104591 (PMC10140157; doi:10.1016/j.jbc.2023.104591)
Supplement: Supporting information [file mmc1.docx]

**Molecular Determinants for Differential Activation of the *Vibrio parahaemolyticus* Bile Acid Receptor**

Angela J. Zou^1^, Lisa Kinch^2^, Suneeta Chimalapati^1,3^, Nalleli Garcia^1,4^, Diana R. Tomchick^5^, Kim Orth^1,2,*^

^1^Department of Molecular Biology, University of Texas Southwestern Medical Center, Dallas, TX 75390, USA

^2^Department of Biochemistry, University of Texas Southwestern Medical Center, Dallas, TX 75390, USA

^3^Howard Hughes Medical Institute, University of Texas Southwestern Medical Center, Dallas, TX 75390, USA

^4^Department of Microbiology and Cell Science, University of Florida Institute of Food and Agricultural Sciences, Gainesville, FL 32603, USA

^5^Department of Biophysics, University of Texas Southwestern Medical Center, Dallas, TX 75390, USA

*Correspondence: [kim.orth@utsouthwestern.edu](mailto:kim.orth@utsouthwestern.edu)

**Supporting Information included:**

Tables S1, S2, S3, S4, S5, S6 Figures S1, S2, S3, S4, S5, S6, S7

**Supporting Information**

**Table S1.** Mean B-values (Å^2^) for each heterodimer in the CDC and TDC crystals.

| **Crystal** | **Native + CDC Mean B-values (Å^2^)** | | **Native + TDC* Mean B-values (Å^2^)** | |
| --- | --- | --- | --- | --- |
| **Heterodimer chain ID** | **VtrA** | **VtrC** | **VtrA** | **VtrC** |
| A:B | 24.6 | 32.1 | 25.7 | 29.8 |
| C:D | 27.4 | 33.6 | 34.9 | 37.8 |
| E:F | 48.3 | 63.1 | 39.1 | 36.2 |
| G:H | 68.5 | 70.1 | - | - |

*Values for PDB ID: 5KEW[7].

**Table S2.** Thermodynamic parameters with 68.3% confidence intervals (CI) of TDC and CDC binding to various VtrA/VtrC constructs measured by ITC.

| **VtrC Construct** | **Bile Acid** | **K_D_ (nM)** | **68.3% CI (nM)** | **ΔH (kcal/mol)** | **68.3% CI (kcal/mol)** |
| --- | --- | --- | --- | --- | --- |
| WT | TDC | 129.4 | (87.6, 186.7) | -11.4 | (-11.9, -10.9) |
| H50A | TDC | 301.2 | (249.8, 360.9) | -11.7 | (-12.1, -11.4) |
| Y81A | TDC | 1,189.1 | (834.5, 1,688.6) | -6.6 | (-7.5, -6.0) |
| S123A | TDC | 91.2 | (49.0, 161.0) | -9.1 | (-9.6, -8.7) |
| Y151A | TDC | 2,499.7 | (1,687.8, 3,890.2) | -11.6 | (-13.1, -10.4) |
| Y151F | TDC | 873.2 | (703.0, 1,079.0) | -10.2 | (-10.8, -9.7) |
|  |  |  |  |  |  |
| WT | CDC | 310.3 | (236.8, 402.7) | -14.0 | (-14.8, -13.3) |
| H50A | CDC | 904.5 | (785.2, 1,042.4) | -9.2 | (-9.6, -8.9) |
| Y81A | CDC | 730.7 | (615.4, 866.2) | -7.8 | (-8.2, -7.4) |
| S123A | CDC | 202.1 | (146.3, 273.7) | -13.9 | (-14.6, -13.2) |
| Y151A | CDC | 2,282.9 | (1,954.1, 2,683.5) | -13.9 | (-14.8, -13.1) |
| Y151F | CDC | 1,166.3 | (979.2, 1,390.9) | -9.6 | (-10.1, -9.2) |

**Table S3.** Thermodynamic parameters with 68.3% confidence intervals (CI) of various bile acids binding to VtrA/VtrC wild-type as measured by ITC.

| **Bile Acid** | **K_D_ (nM)** | **68.3% CI (nM)** | **ΔH (kcal/mol)** | **68.3% CI (kcal/mol)** |
| --- | --- | --- | --- | --- |
| TDC | 129.4 | (87.6, 186.7) | -11.4 | (-11.9, -10.9) |
| DC | 232.0 | (178.9, 297.5) | -13.3 | (-13.7, -12.9) |
| GCDC | 211.4 | (159.0, 277.3) | -13.2 | (-13.7, -12.7) |
| CA | 377.1 | (320.6, 442.1) | -13.3 | (-13.7, -12.9) |
| CDC | 310.3 | (236.8, 402.7) | -14.0 | (-14.8, -13.3) |

**Table S4.** Thermodynamic parameters with 68.3% confidence intervals (CI) of TDC and CDC binding to VtrA/VtrC constructs under different pH conditions measured by ITC.

| **VtrC + Bile Acid** | **pH** | **K_D_ (nM)** | **68.3% CI (nM)** | **ΔH (kcal/mol)** | **68.3% CI (kcal/mol)** |
| --- | --- | --- | --- | --- | --- |
| WT + TDC | 7 | 95.1 | (72.8, 122.0) | -11.8 | (-12.1, -11.5) |
| WT + TDC | 8 | 129.4 | (87.6, 186.7) | -11.4 | (-11.9, -10.9) |
| WT + TDC | 9 | 176.2 | (119.2, 252.2) | -10.6 | (-11.1, -10.1) |
|  |  |  |  |  |  |
| H50A + TDC | 7 | 414.2 | (316.7, 539.0) | -13.0 | (-13.6, -12.4) |
| H50A + TDC | 8 | 301.2 | (249.8, 360.9) | -11.7 | (-12.1, -11.4) |
| H50A + TDC | 9 | 416.8 | (326.0, 529.8) | -10.2 | (-9.6, -10.7) |
|  |  |  |  |  |  |
| WT + CDC | 7 | 304.9 | (219.0, 418.3) | -13.4 | (-14.4, -12.5) |
| WT + CDC | 8 | 310.3 | (236.8, 402.7) | -14.0 | (-14.8, -13.3) |
| WT + CDC | 9 | 240.5 | (182.1, 314.6) | -11.8 | (-12.3, -11.3) |
|  |  |  |  |  |  |
| H50A + CDC | 7 | 1,922.8 | (1,340.2, 2,808.6) | -9.7 | (-10.6, -8.9) |
| H50A + CDC | 8 | 904.5 | (785.2, 1,042.4) | -9.2 | (-9.6, -8.9) |
| H50A + CDC | 9 | 970.3 | (725.6, 1,299.2) | -7.8 | (-8.6, -7.2) |

**Table S5.** Primers used in this study.

| **Vector** | **Primer Name** | **Primer Sequence** |
| --- | --- | --- |
| pDM4 vtrB-3XFLAG | F/upVtrB_SpeI | AAAAACTAGTGAGTGAAGTCCCTGAGCTTG |
|  | Lf/vtrB_3XFLAG | CTTTATAATCACCGTCATGGTCTTTGTAGTCTTTAAGCAACAAAATCATCGCCGTACTGGTAAC |
|  | Ri/vtrB_3XFLAG | ATCATGACATCGATTACAAGGATGACGATGACAAGTAAACATTGAAAGGCACTAGAAAGGCAGAGTATCT |
|  | R/dnVtrB_SalI | AAAAGTCGACTCCGTAAAAACTTCGTTTATTTCCTAAATATAG |
| pRU1701 vtrB -300bp | vtrb-prom_F_SalI | TAGTCGACaggctaaaggggctatgc |
|  | vtrb-prom_R_SpeI | TATACTAGTcgctgagcccttttcacag |
| pBAD-FLAG-vtrC Q42A | From Li et al., 2016 | |
|  |  |  |
| pACYC-Duet-VtrC/VtrA VtrC H50A pBAD-FLAG-vtrC H50A | VtrC H50A F | TTAATGGCTACGTCAGCCATATATGTGCTGTCAGCGGCC |
|  | VtrC H50A R | GGCCGCTGACAGCACATATATGGCTGACGTAGCCATTAA |
| pACYC-Duet-VtrC/VtrA VtrC Y81A pBAD-FLAG-vtrC Y81A | VtrC Y81A F | GTATAATATCACCATGCCCTATTACATTAGCATAGTTTTTATTCTCACTTTTTACTAATTCTCTT |
|  | VtrC Y81A R | AAGAGAATTAGTAAAAAGTGAGAATAAAAACTATGCTAATGTAATAGGGCATGGTGATATTATAC |
| pACYC-Duet-VtrC/VtrA VtrC S123A pBAD-FLAG-vtrC S123A | VtrC S123A F | CATGAAACCATATAAAGAGCCGACAGCCATATCTAGTCTGATAAAT |
|  | VtrC S123A R | ATTTATCAGACTAGATATGGCTGTCGGCTCTTTATATGGTTTCATG |
| pACYC-Duet-VtrC/VtrA VtrC Y151A pBAD-FLAG-vtrC Y151A | VtrC Y151A F | TTATTATTTGTCCATCAGCAAAGAAAAACTCCACAACCACGTACTCTTCGGA |
|  | VtrC Y151A R | TCCGAAGAGTACGTGGTTGTGGAGTTTTTCTTTGCTGATGGACAAATAATAA |
| pACYC-Duet-VtrC/VtrA VtrC Y151F pBAD-FLAG-vtrC Y151F | VtrC Y151F F | GAAGAGTACGTGGTTGTGGAGTTTTTCTTTTTTGATGGACAAATAATA |
|  | VtrC Y151F R | TATTATTTGTCCATCAAAAAAGAAAAACTCCACAACCACGTACTCTTC |

**Table S6.** Data collection and refinement statistics, VtrA/C and CDC complex

| Space group | C2 |
| --- | --- |
| Cell constants (Å, °) | a = 142.60, b = 41.76, c = 168.96, β = 91.57° |
| Wavelength (Å) | 0.97926 |
| Resolution range (Å) | 44.78 – 2.08 (2.12 – 2.08) |
| Unique reflections | 57,573 (2,115) |
| Multiplicity | 3.4 (1.7) |
| Data completeness (%) | 94.5 (70.4) |
| *R*_merge_ (%)^a^ | 8.7 (27.2) |
| *R*_pim_ (%)^b^ | 5.0 (22.4) |
| CC_1/2_ (last resolution shell) | 0.876 |
| I/σ(I) | 14.9 (2.5) |
| Wilson *B*-value (Å^2^) | 25.8 |
| Resolution range (Å) | 44.78 – 2.08 (2.13 – 2.08) |
| No. of reflections *R*_work_/R_free_ | 54,304/2,007 (2,054/77) |
| Data completeness (%) | 88.9 (50.0) |
| Atoms (non-H protein/ions/ligands/solvent) | 7,370/4/112/345 |
| *R*_work_ (%) | 18.8 (22.7) |
| *R*_free_ (%) | 22.4 (23.3) |
| R.m.s.d. bond length (Å) | 0.010 |
| R.m.s.d. bond angle (°) | 0.78 |
| Mean B-value (Å^2^) (protein chain ID) (ions/ligands/solvent) | A: 24.6; B: 32.1; C: 27.4; D: 33.6; E: 48.3; F: 63.1; G: 68.5; H: 70.1/57.4/45.5/40.1 |
| Ramachandran plot (%) (favored/additional/disallowed)^c^ | 96.44/3.45/0.11 |
| Clashscore/Overall score^c^ | 3.33 |
| Maximum likelihood coordinate error | 0.23 |
| Missing residues | A: 161 – 164. B: -12 – -2. C: 161 – 163. D: -12 – -2. E: 161 – 164. F: -12 – -1. G: 161 – 164. H: -12 – -1. |

Data for the outermost shell are given in parentheses.

^a^*R*_merge_ = 100 Σ_h_Σ_i_|*I_h,i_*— 〈*I_h_*〉*|/*Σ*_h_*Σ_i_ 〈*I_h,i_*〉, where the outer sum (h) is over the unique reflections and the inner sum (i) is over the set of independent observations of each unique reflection.

^b^*R*_pim_ = 100 Σ_h_Σ_i_ [1/(n_h_ - 1)]^1/2^|*I_h,i_*— 〈*I_h_*〉*|/*Σ*_h_*Σ_i_ 〈*I_h,i_*〉, where n_h_ is the number of observations of reflections **h**.

^c^As defined by the validation suite MolProbity [28].


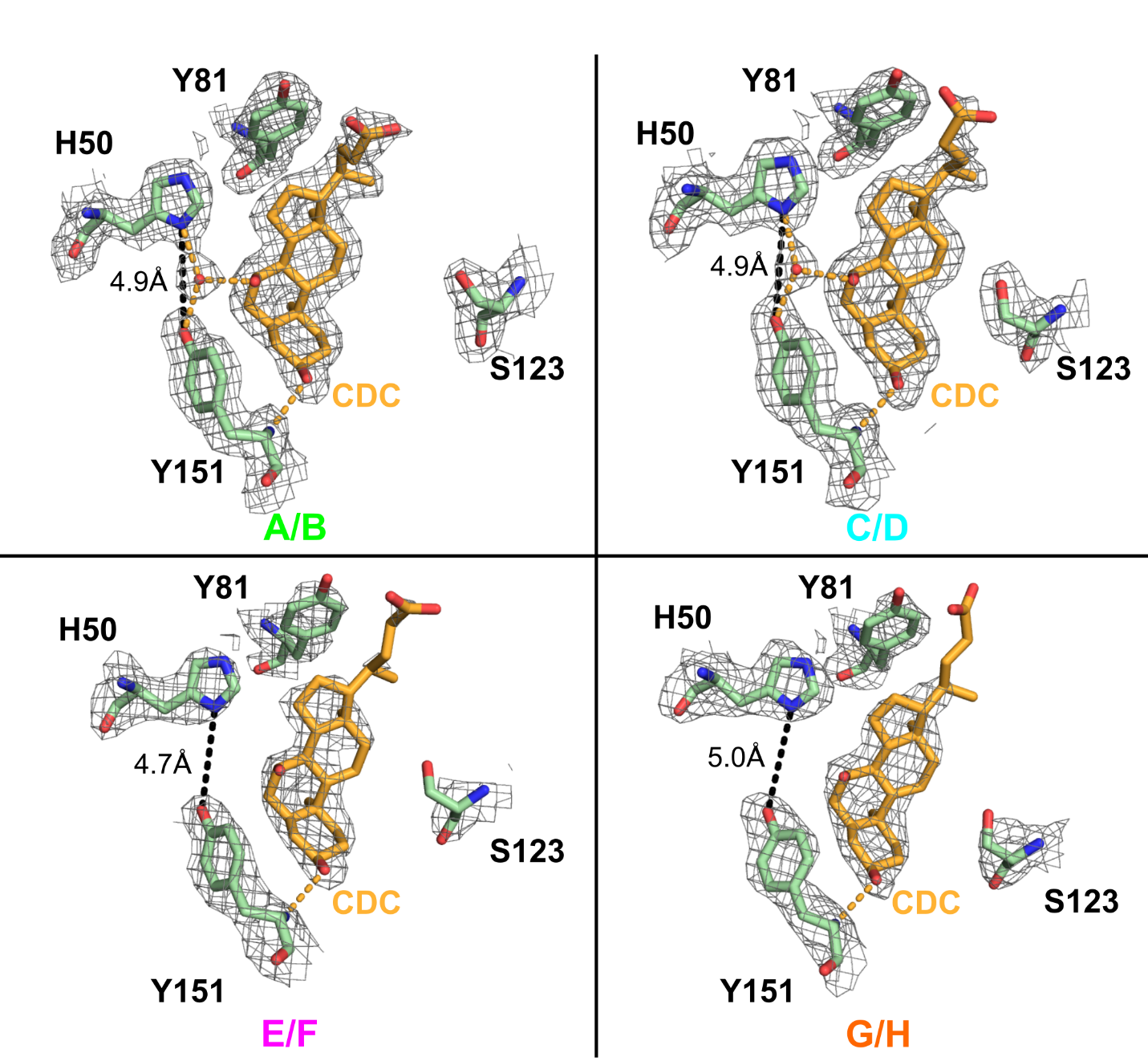


**Figure S1. Electron density around CDC molecules and binding pocket residues.** Kicked F_o_-F_c_ omit maps of the four CDC molecules bound to the A/B, C/D, E/F, and G/H heterodimers (indicated in green, cyan, magenta, and orange). CDC molecules (orange) and binding pocket residues (light green) are shown as sticks. Modeled water molecules are shown as red spheres. Hydrogen bonds are shown as orange dashed lines. Distance measurements are indicated with black dashed lines. Maps are shown as grey mesh (contoured at the 1σ level) and carved around sticks at a 1.6 Å radius.


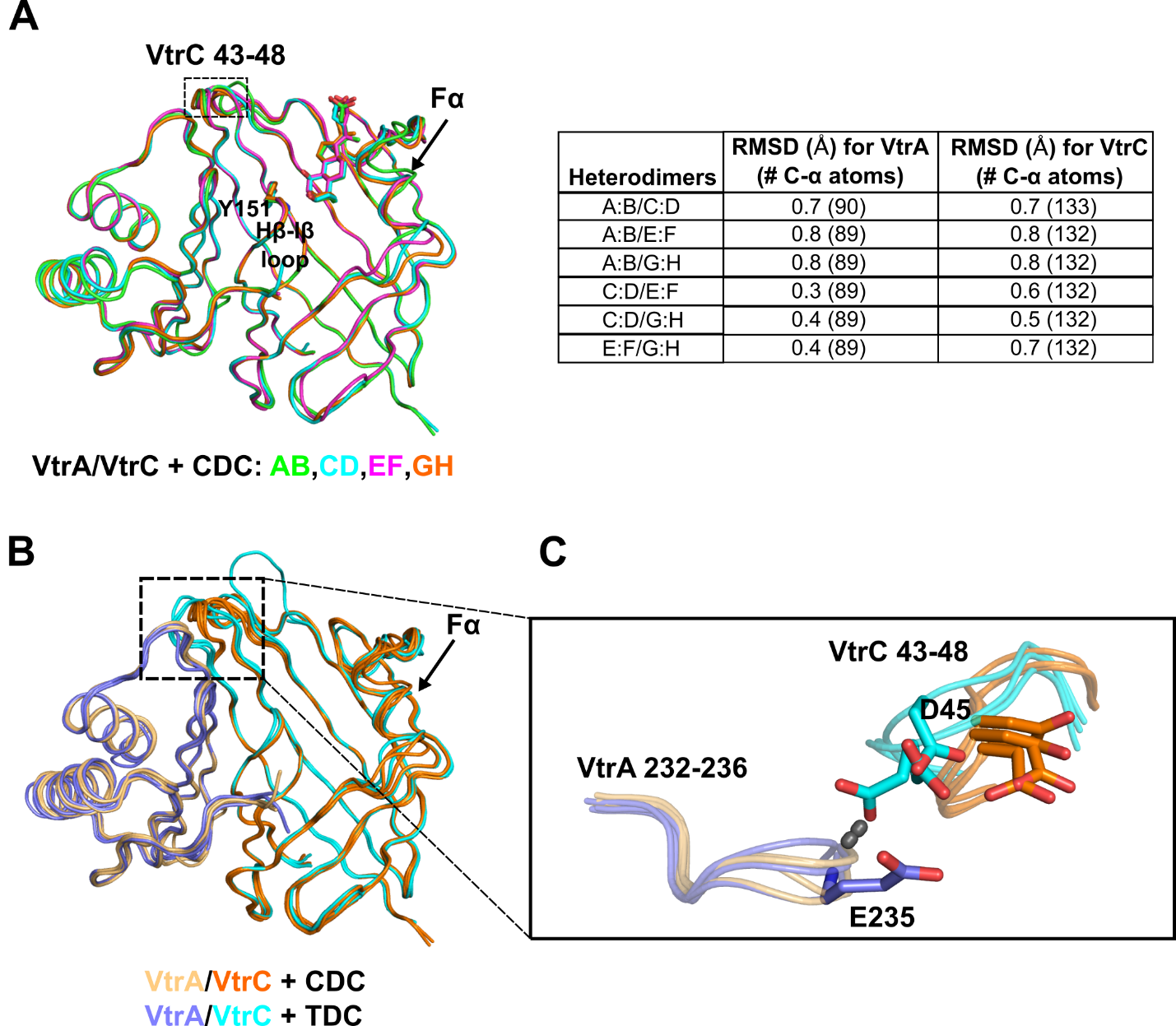


**Figure S2. Alignment of the CDC-bound structure heterodimers and comparison with TDC-bound structure heterodimers.** **(A)** (Left) Superposition of ribbon models for all four heterodimers in the asymmetric unit of the CDC-bound crystal. VtrC Y151 side chain and CDC shown as sticks. (Right) Root-mean-square-deviations (RMSD) for each superposition in (Å) with the number of aligned α-carbon atoms in parentheses. Superpositions were made via the DaliLite server (<http://ekhidna.biocenter.helsinki.fi/dali_lite/start>) [33]. **(B)** Superposition of (A) with the three heterodimers in the asymmetric unit of the TDC-bound crystal as ribbons. CDC-bound VtrA/VtrC are colored in shades of orange. TDC-bound VtrA/VtrC are colored in shades of blue. **(C)** “Top” view of VtrA/VtrC interface boxed in (B). VtrA/VtrC modelled as ribbons. VtrA E235 and VtrC D45 side chains shown as sticks. Hydrogen bond shown as a grey dashed line.

**
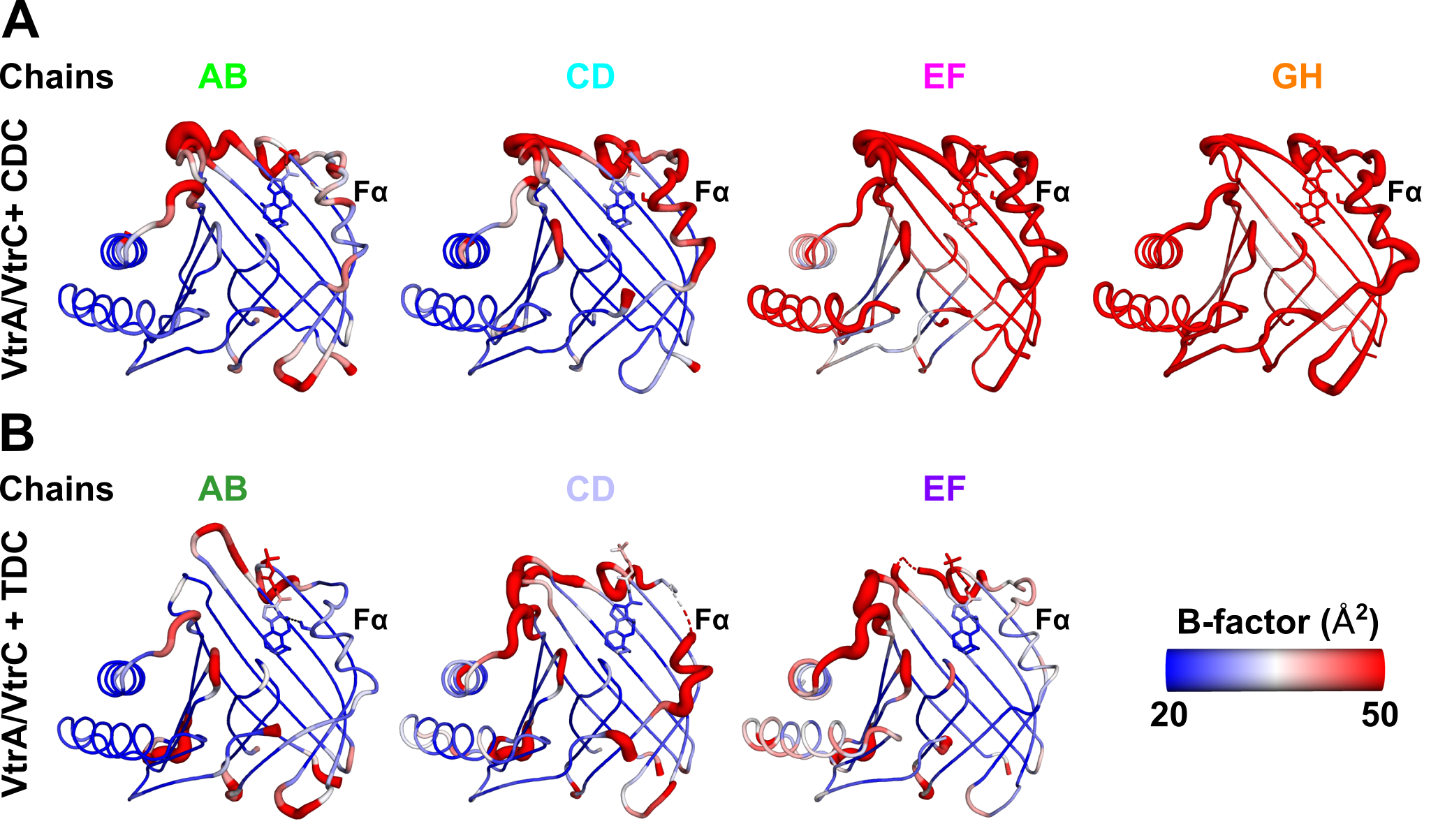
**

**Figure S3. Representation of B-factors in each heterodimer of the CDC- and TDC-bound crystals.** **(A)** The structure of each CDC-bound heterodimer is represented as cartoon putty and colored based on the B-factors of each residue. Fα helix is labeled for each heterodimer. (**B)** B-factors of each TDC-bound heterodimer in crystal 5KEW[7]. B-factor color scale shown in the rightmost panel.


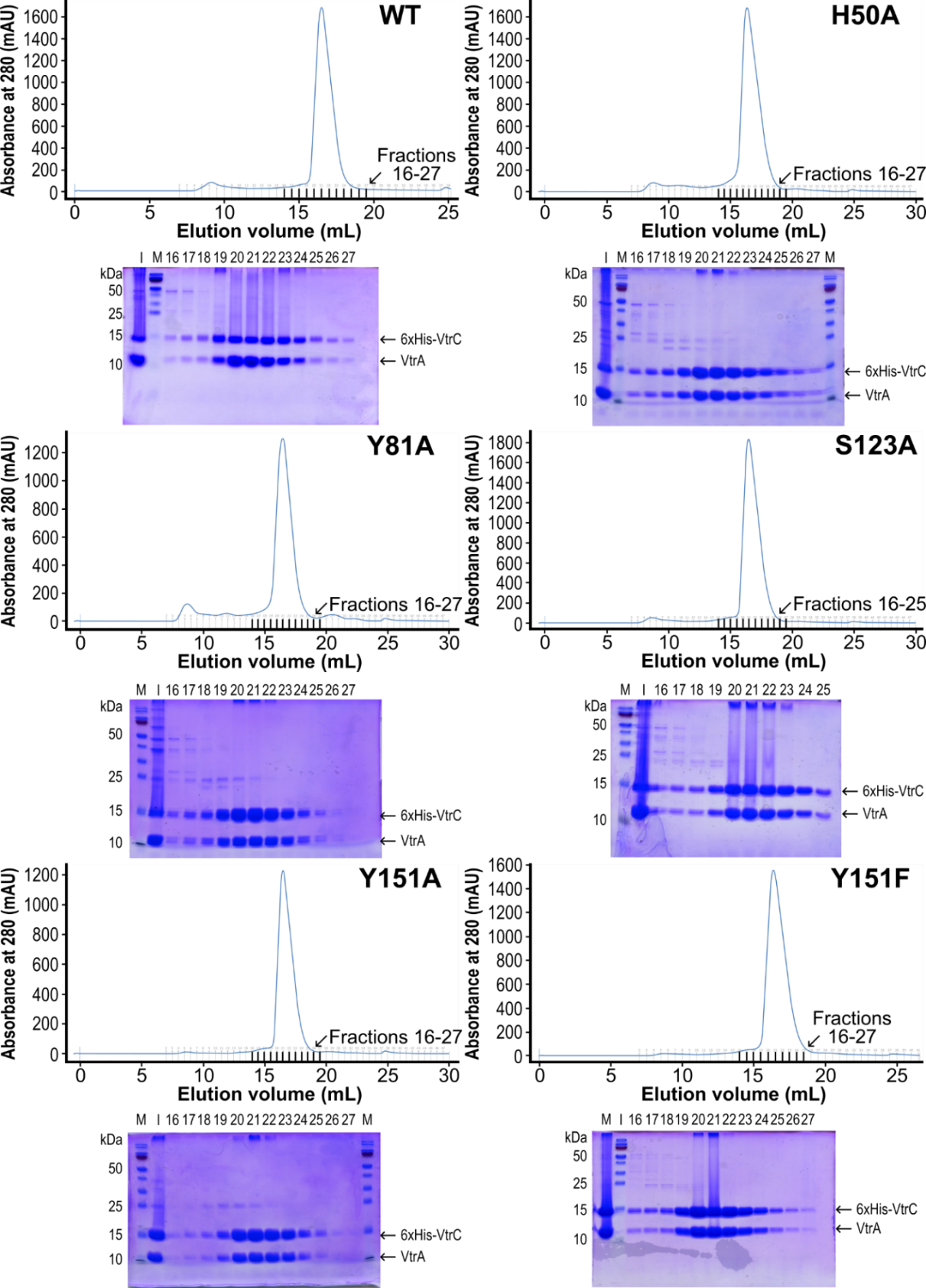


**Figure S4. VtrC wild-type and mutant periplasmic domain constructs all bind to the VtrA periplasmic domain in solution.** A280 absorbance (mAU) gel filtration profiles of each VtrA/VtrC construct. Elution volume (mL) is plotted on the x-axis. Peak fractions are labeled and indicated with black lines. Under each profile is the Coomassie gel of the protein MW ladder (M), input (I) and numbered peak fractions for each construct. Protein bands for 6xHis-VtrC and untagged VtrA are indicated with arrows.


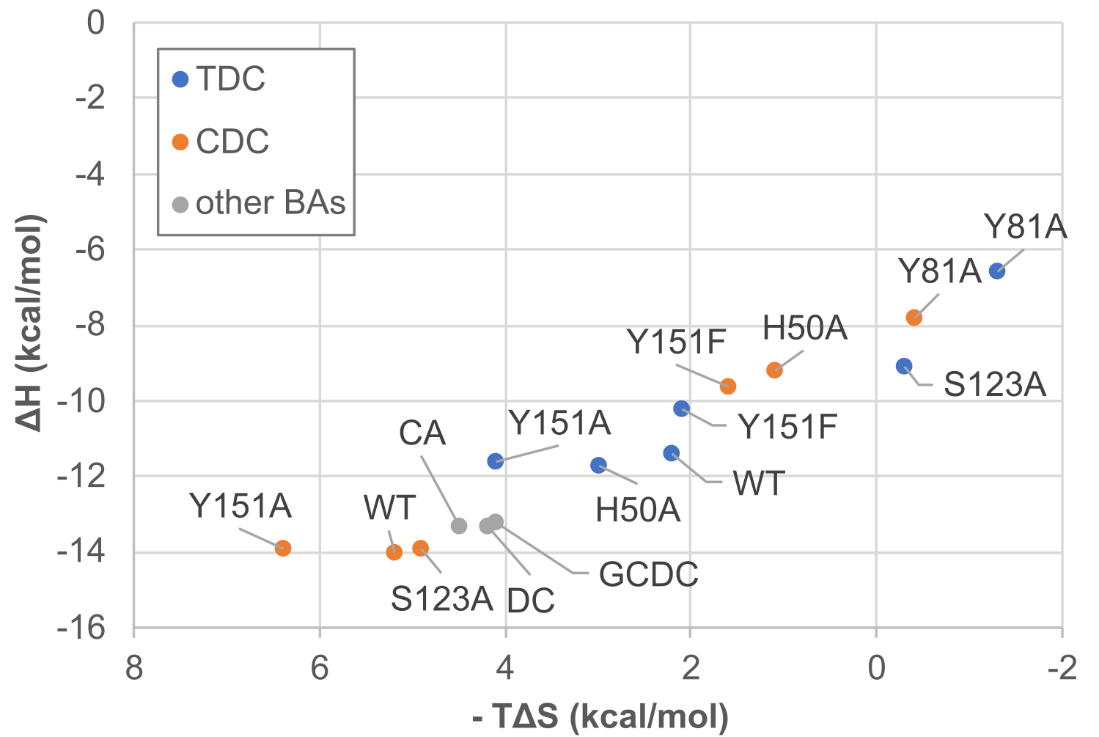


**Figure S5. Enthalpy-entropy compensation of bile acid binding to VtrA/VtrC constructs.** Plot of the ΔH (kcal/mol) vs. -TΔS (kcal/mol) values determined by ITC for TDC (blue dots) and CDC (orange dots) binding to VtrA/VtrC wild-type and mutants. (Grey dots) binding of the bile acids CA, DC, and GCDC (other BAs) to wild-type VtrA/VtrC.

**
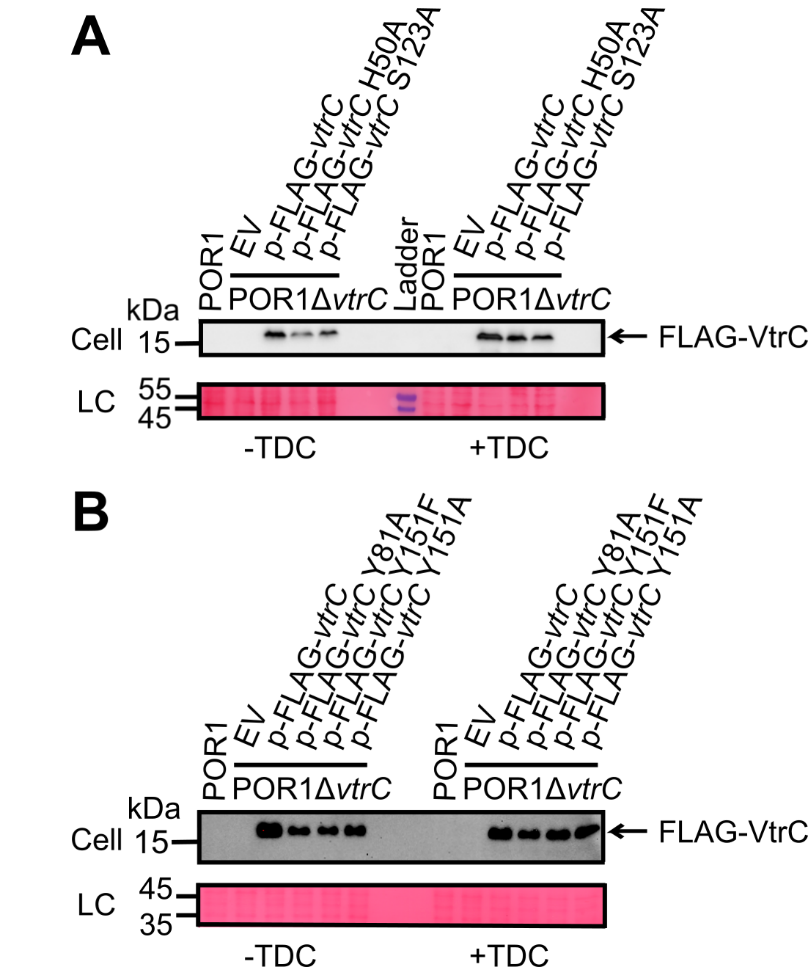
**

**Figure S6. Expression of FLAG-*vtrC* mutants.** **(A,B)** Expression (Cell) of FLAG-tagged VtrC in POR1 and POR1Δ*vtrC* strains containing an empty pBAD vector (EV) or pBAD FLAG-*vtrC* variants (p-FLAG-*vtrC*). Anti-FLAG antibody (Sigma) was used to detect FLAG-tagged VtrC. -/+ TDC, *V. parahaemolyticus* grown in MLB without TDC (-TDC) or supplemented with 100 μM TDC (+TDC). Loading control (LC) is shown for total cell lysate.

**
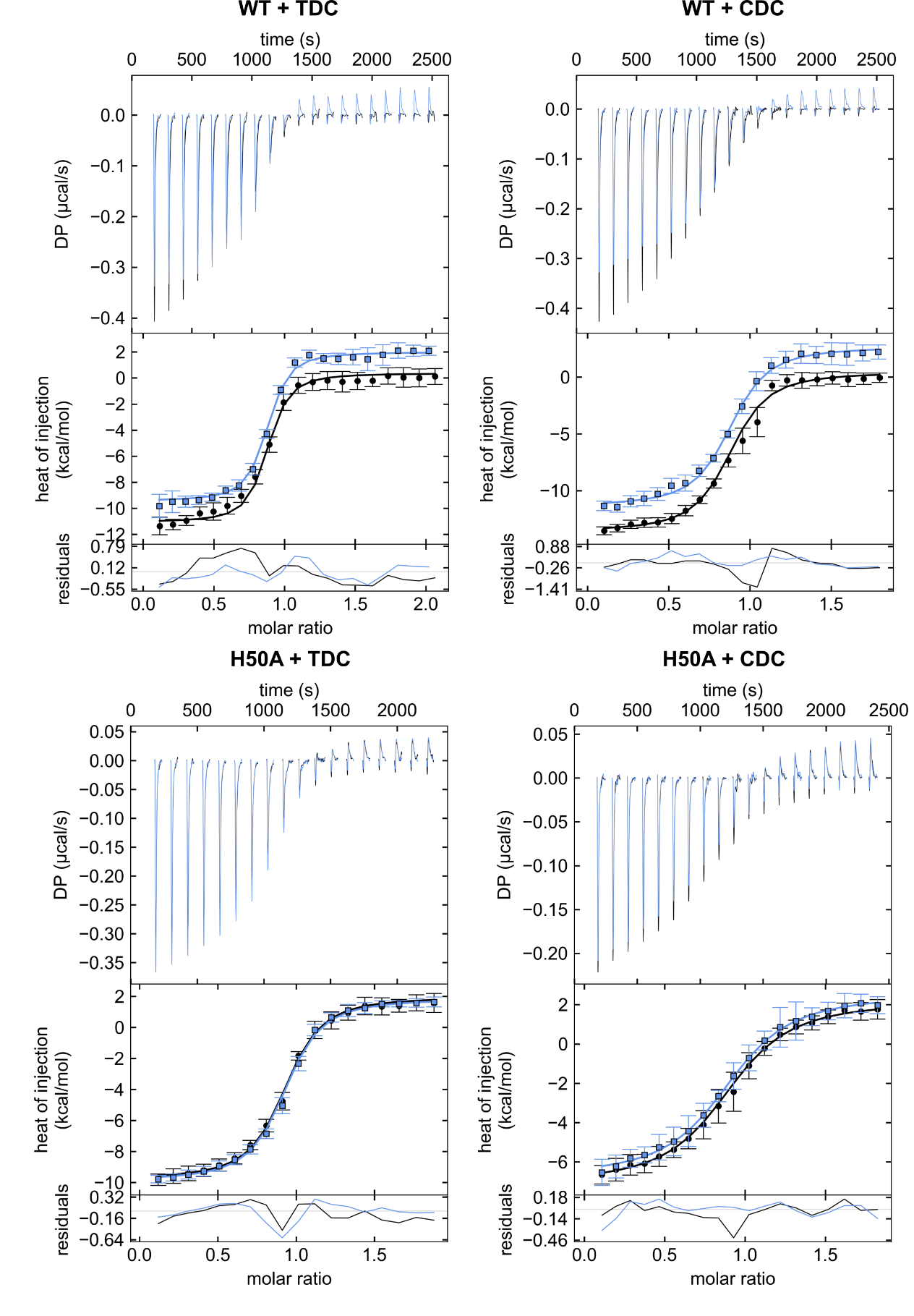
**

**Figure S7.** **ITC thermograms for binding of TDC and CDC to VtrA/VtrC constructs.** Thermodynamic parameters (Table 1, Table S2) were determined by global fitting of duplicate isotherms (presented in black and blue).


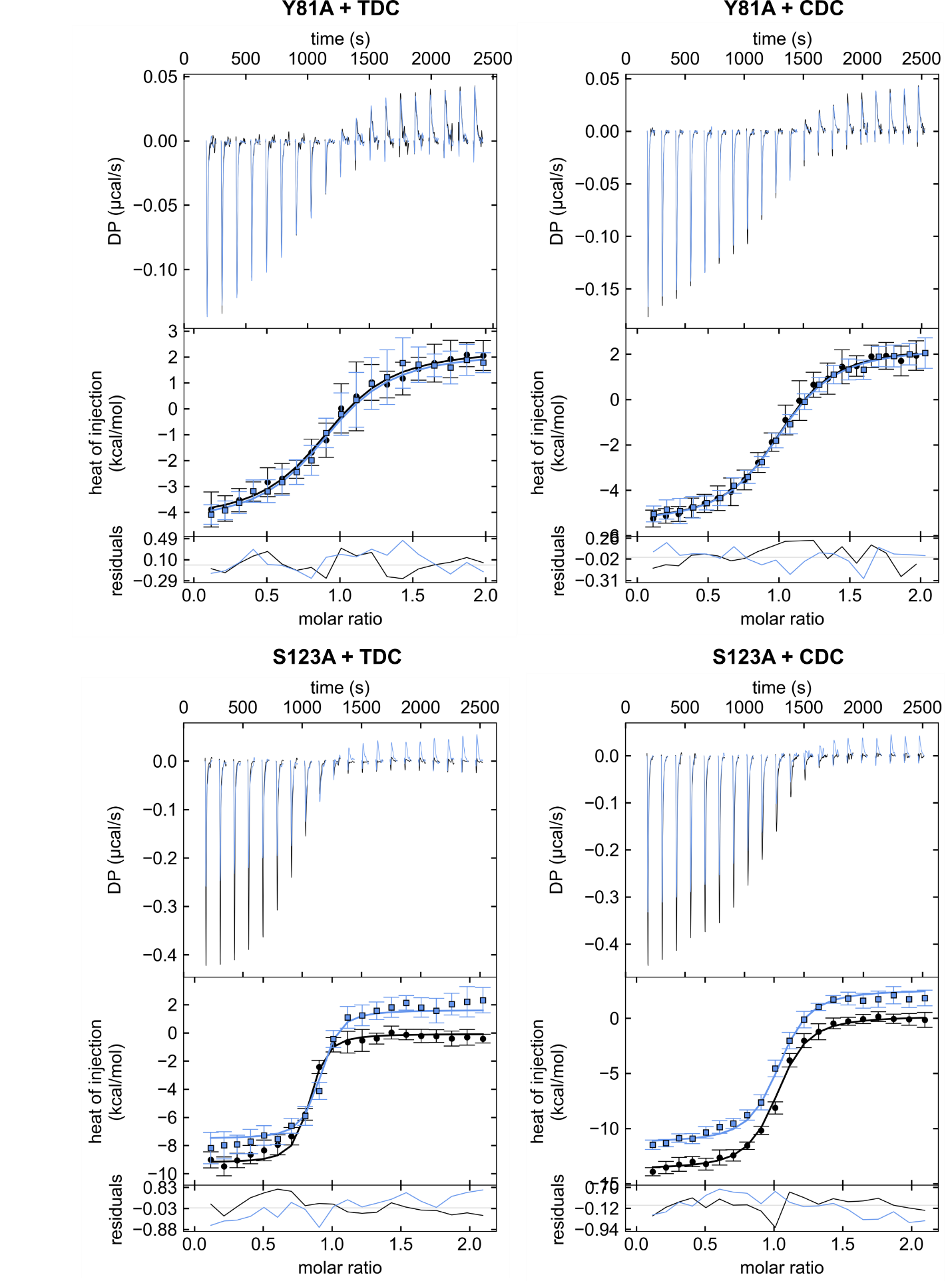


**Figure S7 (continued)**

**
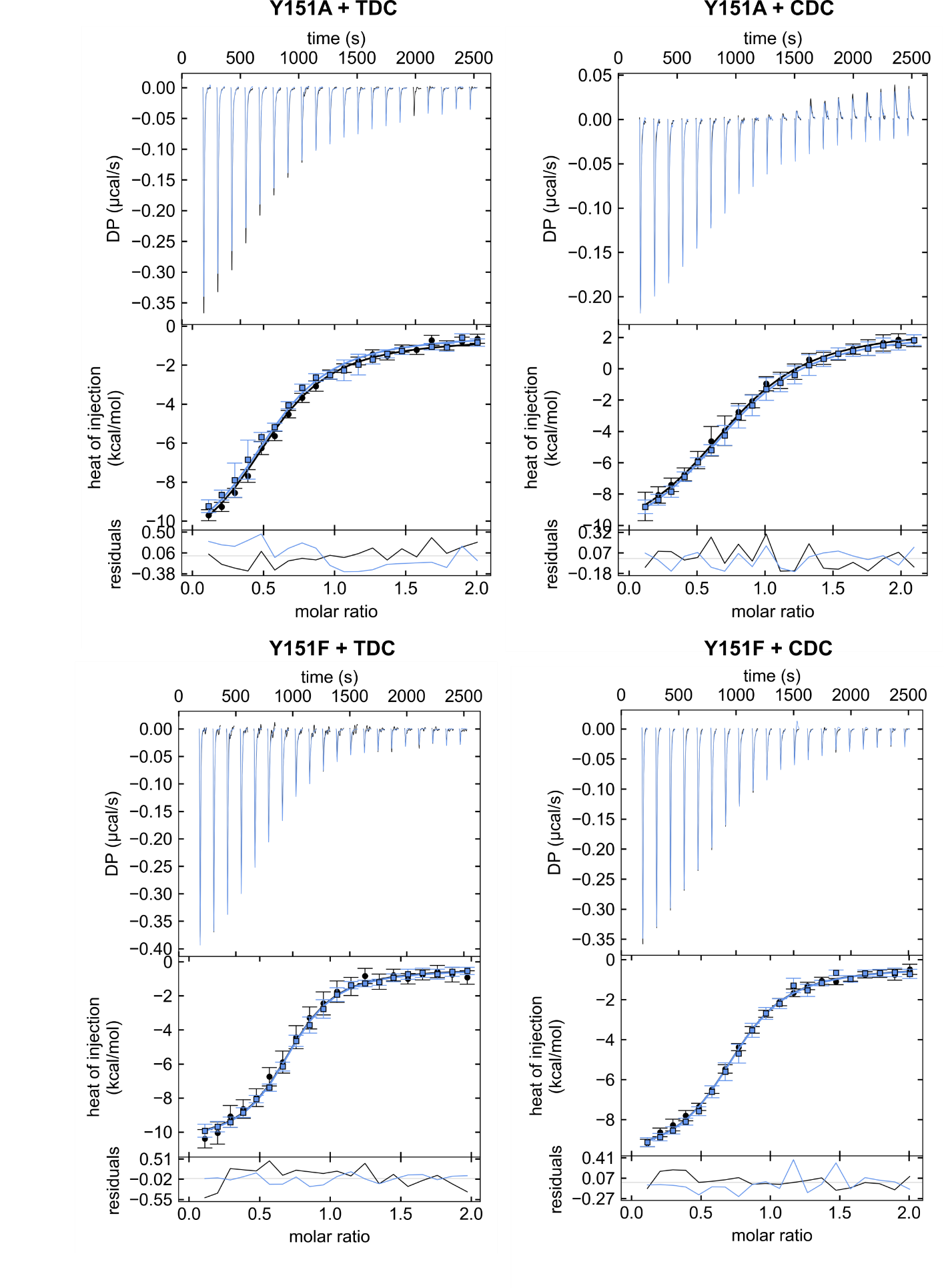
**

**Figure S7 (continued)**
